# Global status and trends of gastric cancer and gastric microbiota research: a bibliometric analysis

**DOI:** 10.3389/fmicb.2024.1341012

**Published:** 2024-04-09

**Authors:** Yujia Ke, Cheng Tan, Junhai Zhen, Weiguo Dong

**Affiliations:** ^1^Department of Gastroenterology, Renmin Hospital of Wuhan University, Wuhan, Hubei, China; ^2^Department of General Practice, Renmin Hospital of Wuhan University, Wuhan, Hubei, China

**Keywords:** gastric cancer, stomach microbiota, gastric carcinogenesis, bibliometric, CiteSpace

## Abstract

**Background:**

Numerous studies have cast light on the relationship between the gastric microbiota and gastric carcinogenesis. In this study, we conducted a bibliometric analysis of the relevant literature in the field of gastric cancer and the gastric microbiota and clarified its research status, hotspots, and development trends.

**Materials and methods:**

Publications were retrieved from the Web of Science Core Collection on 18 July 2023. CiteSpace 6.2.R4, VOSviewer 1.6.19.0, and Biblioshiny were used for the co-occurrence and cooperation analyses of countries, institutions, authors, references, and keywords. A keyword cluster analysis and an emergence analysis were performed, and relevant knowledge maps were drawn.

**Results:**

The number of published papers in this field totaled 215 and showed an increasing trend. The analysis of funding suggested that the input in this field is increasing steadily. China had the highest number of publications, while the United States had the highest betweenness centrality. Baylor College of Medicine published the most articles cumulatively. Both Ferreira RM and Cooker OO had the highest citation frequency. The journal *Helicobacter* showed the most interest in this field, while *Gut* provided a substantial research foundation. A total of 280 keywords were obtained using CiteSpace, which were primarily focused on the eradication and pathogenic mechanisms of *Helicobacter pylori*, as well as the application of the gastric microbiota in the evaluation and treatment of gastric cancer. The burst analysis suggested that in the future, research may focus on the application of gastric microorganisms, particularly *Fusobacterium nucleatum*, in the diagnosis and treatment of gastric cancer, along with their pathogenic mechanisms.

**Conclusion:**

Current studies have been tracking the eradication of *Helicobacter pylori* and its pathogenic mechanisms, as well as changes in the gastric microbiota during gastric carcinogenesis. Future research may focus on the clinical application and pathogenesis of stomach microorganisms through bacteria such as *Fusobacterium nucleatum*.

## 1 Introduction

Gastric cancer (GC) is the fifth most common cancer in the world and the fourth leading cause of cancer death (Sung et al., 2021). In the future, although the incidence rate of GC may show a downward trend, for some countries, incidence and mortality increases have been predicted in people below the age of 50 (Arnold et al., 2020; Qi et al., 2023; Teng et al., 2023). *Helicobacter pylori* (*H. pylori*) is considered a major risk factor for GC and has been classified as a Class I carcinogen. The birth-cohort pattern revealed an epidemic of *H. pylori* in gastrointestinal disease (Sonnenberg, 2022). The sequential development model of GC suggests that *H. pylori* colonizes the gastric mucosa, inducing continuous chronic gastric inflammation, then causes cascade pathogenesis: atrophic gastritis (AG), in which *H. pylori* plays a role, and then intestinal metaplasia (IM), dysplasia, and finally GC (Correa, 1988, 1992). However, the decreasing prevalence of *H. pylori* has been histologically observed with the increasing severity of AG (Correa, 1992; Kuipers, 1998; Liu et al., 2022), and some clinical studies have shown that *H. pylori* eradication in patients with advanced lesions does not eliminate the risk of carcinogenesis (Wong et al., 2004; Rugge et al., 2019), indicating that further progression of precancerous conditions may be independent of *H. pylori* colonization. With the prevalence of *H. pylori* infection decreasing (Li et al., 2023), these facts draw attention to gastric microorganisms other than *H. pylori*.

The gastrointestinal microbiota is the largest and most complex microbial ecosystem in the human body, among which bacteria form major communities. However, given its highly acidic environment, which makes it difficult for general bacteria to colonize, the stomach was considered sterile until *H. pylori* was isolated from the gastric mucosa. With the development of molecular techniques, 128 phylotypes of microorganisms have been identified through phylogenetic analysis from gastric endoscopy biopsy samples, most of which belong to *Proteobacteria*, *Firmicutes*, *Bacteroidetes*, *Actinobacteria*, and *Fusobacteria*, and 10 main genera have been classified (Bik et al., 2006; Liu et al., 2022). Multiple case–control studies based on gastric mucosal tissue biopsies have confirmed changes in bacterial diversity during the progression of intestinal-type GC. From non-atrophic gastritis (NAG) to IM and then to GC, the bacterial diversity steadily decreased (*p* = 0.004), and the gastric flora abundance changed continuously during this process (Aviles-Jimenez et al., 2014; Liu et al., 2022). Some studies have put forward different views: compared with functional dyspepsia, the richness and diversity of bacterial flora in GC tissue increased, but their uniformity did not increase. In addition, the serological status of *H. pylori* has a significant impact on the composition and diversity of the gastric microbiome (Castano-Rodriguez et al., 2017). Evidence from germ-free insulin–gastrin (INS-GAS) and human gastric microbiota transplant mouse models further supports the potential causality of the microbiota in gastric carcinogenesis (Lofgren et al., 2011; He C. et al., 2022; Kwon et al., 2022). Accordingly, some researchers have used combinations of genera of gastric flora as microbial markers for the non-invasive diagnosis of GC (Chen et al., 2023).

Bibliometrics combines mathematics, statistics, and literature to explore the structural characteristics and hot trends of disciplines through the quantitative analysis of vast amounts of publications and evaluates and predicts the results. It has been widely applied in various fields of medicine (Dracos and Cognetti, 1995; Chen et al., 2012; Kokol et al., 2021). Compared to traditional systematic reviews, the application of bibliometrics in a research field can help new researchers or researchers in other fields to grasp the development process and status of the field based on cluster labels or topics, rather than reading hundreds of unfamiliar studies to obtain limited information. It might also be limited to reading reviews because they often focus on only one research theme (Donthu et al., 2021). For small data samples, synthetic knowledge synthesis can also be applied to extract, synthesize, and multidimensionally structure the corpus of scholarship (Kokol et al., 2022). At present, scholars are increasingly reporting on GC and the gastric microbiota. However, to the best of our knowledge, there is currently no intuitive visual analysis that explores the hotspots and trends in this field. Therefore, we adopted bibliometric methods to conduct a systematic review of this field. By performing quantitative and qualitative analyses of the relevant literature and utilizing visualization tools, the research status, hotspots, and future trends in this field were analyzed.

## 2 Materials and methods

### 2.1 Data source

Data were acquired from the Web of Science Core Collection (WoSCC) on 18 July 2023. The query was ((TS = (“gastric cancer*”) OR TS = (“gastric neoplasm*”) OR TS = (“gastric malignancy”) OR TS = (“gastric adenocarcinoma”) OR TS = (“gastric carcinoma”) OR TS = (“stomach cancer*”) OR TS = (“stomach neoplasm*”) OR TS = (“stomach malignancy”) OR TS = (“stomach adenocarcinoma”) OR TS = (“stomach carcinoma”)) AND (TS = (“gastric microbiota*”) OR TS = (“gastric microbiome*”) OR TS = (“gastric microflora”) OR TS = (“gastric flora”) OR TS = (“gastric bacteria”) OR TS = (“gastric microbial community”) OR TS = (“gastric bacterial community”) OR TS = (“stomach microbiota*”) OR TS = (“stomach microbiome*”) OR TS = (“stomach microflora”) OR TS = (“stomach flora”) OR TS = (“stomach bacteria”) OR TS = (“stomach microbial community”) OR TS = (“stomach bacterial community”))). The language was limited to English. Two evaluators screened the literature independently by reading the titles and keywords, and differences were settled through discussion. A third researcher would participate in further discussion, and a consensus would be reached if the dispute was still inconclusive.

### 2.2 Data creation and statistical analysis

#### 2.2.1 Data collection and transformation

To export the retrieved documents, “full record and cited references” was selected. Data were converted to “txt” or “csv” format, named “download_*.txt,” and then imported into CiteSpace 6.2.R4 and VOSviewer 1.6.19.0 for analysis (Chen, 2006; van Eck and Waltman, 2010). These bibliometric software programs are the most popular and have powerful features. Biblioshiny is a bibliometric program powered by Bibliometrix in the R language. After importing bibliographic records, it can quickly generate visual graphics based on the data, making it a convenient and comprehensive analysis tool (Aria and Cuccurullo, 2017). By using different software, we can achieve complementary functions and verify the analysis results against each other (Cobo et al., 2011; Moral-Munoz et al., 2020).

#### 2.2.2 Data processing

Using Microsoft Excel, the publication volumes of the literature and the funding information were analyzed. CiteSpace 6.2.R4 was applied to deduplicate the obtained documents, retaining only articles and reviews, and then conduct visual analysis. The time span was selected from 2013 to 2023, and the time slice was selected as 1 year. As we described later, we adjusted the k-value appropriately to perform co-occurrence analyses on countries, institutions, and authors and co-citation analyses on journals. Moreover, keywords were used for co-occurrence, clustering, and emergence analyses. Different nodes represent different elements. The color of the ring corresponds to the time when the element appears, the width of the ring represents the frequency of the element at that time, and the size of the whole node reflects the total frequency of the element. The connecting lines between nodes represent co-occurrence, cooperation, or co-citation. The color of the links represents the time when the association first appeared, and its thickness represents the strength of the association. Other parameters were default.

In addition, VOSviewer 1.6.19.0 and Pajek were employed to analyze keyword clusters. Biblioshiny was used to carry out supplementary analyses of the cooperation network, important citing documents, cited source journals, as well as the frequency of keywords and research topic evolution. It is worth noting that in Biblioshiny, we used Keywords Plus for topic analysis due to missing Author Keywords in some literature. Similarly, we detected Author Keywords and Keywords Plus in CiteSpace and VOSviewer. Keywords Plus is as effective as Author Keywords in terms of bibliometric analysis investigating the knowledge structure but is less comprehensive in representing an article’s content (Zhang et al., 2016). Therefore, we combined the analysis results of different software programs and read the relevant literature to better describe the topics in the field.

#### 2.2.3 Relative statistical indicators and parameters

##### 2.2.3.1 Parameters in CiteSpace

The g-index is a parameter that can better measure the influence of an author. In CiteSpace, it belongs to the selection criteria and is calculated as *g*^2^ ≤ $k\underset{i\leq g}{\sum }c_{i}$ , k∈Z. Therefore, by adjusting the k-value, the number of nodes can be changed. In order to include as many nodes as possible and exclude less important nodes to ensure the reliability of the analysis, we adopted different k-value settings. The k-value was set to 25 in the co-occurrence analysis of countries, institutions, and keywords; 15 in the authors’ co-occurrence analysis and the journal’s co-citation analyses.

Betweenness centrality is one of the main metrics in network analysis. It refers to a node’s ability to carry information between unconnected groups of nodes, wherein each node represents a research constituent (Donthu et al., 2021). If a node has a centrality greater than 0.1, it is a critical node, as shown by the purple outer ring. The higher the centrality, the more important the bridge function of the node in the whole network.

The emergence detection analysis can reflect the development of an element. For the keywords burst detection, we set γ to 0.45 and the minimum duration to 1.

##### 2.2.3.2 Parameters in Biblioshiny

Callon centrality and density are two parameters that determine the position of bubbles. Centrality is the degree of correlation among different topics. The higher the number of relations a node has with others in the thematic network, the higher the centrality and importance are. Density measures the cohesiveness among a node, which represents the theme’s development and delineates its capability to develop and sustain itself (Esfahani et al., 2019; Singh et al., 2023). In the thematic map, density is represented on the vertical axis, while centrality takes the horizontal axis, dividing the map into four quadrants. The upper right quadrant (Q1) contains motor themes, which are important to the research field and have the potential to develop. The upper left quadrant (Q2) contains highly developed and isolated themes, which have abundant internal bonds but less contribution to the development of the field. It means that these themes are potential themes to establish contacts with themes in Q1. The lower left quadrant (Q3) contains emerging or declining themes, which have weak development and are marginal. The lower right quadrant (Q4) contains basic and transversal themes, which have a great value to be discussed in the future.

## 3 Results

### 3.1 Research situation analysis

#### 3.1.1 Analysis of publication volume

**Figure 1:**
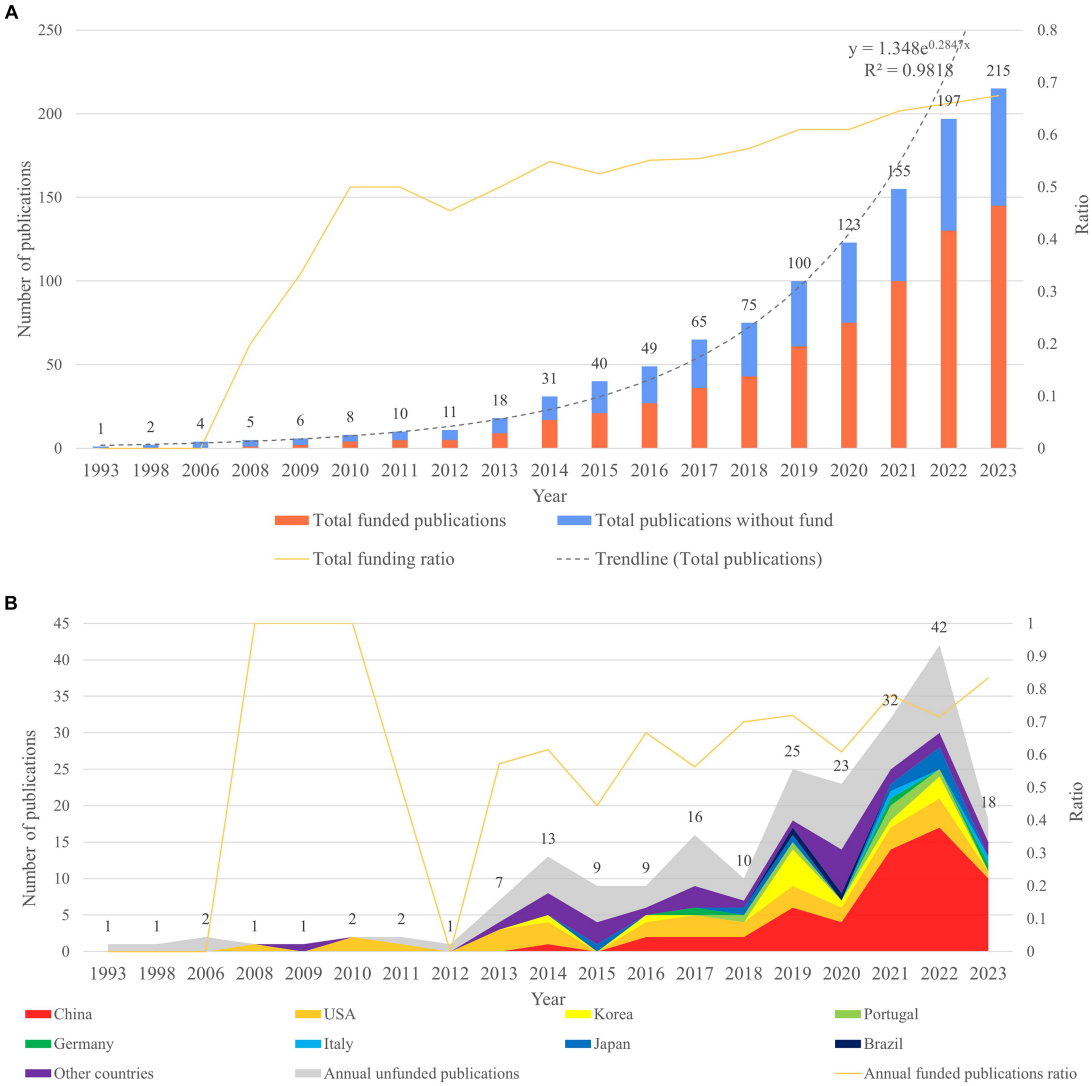
The analysis of publication volume and funding information in the field of gastric microbiota and gastric cancer from 1993 to 2023. **(A)** Total cumulative publication volume and cumulative funding situation productions. **(B)** Annual publication volume and annual funding situation.

Analysis of the number of publications is helpful for initially determining whether a research field has received continued attention from researchers and whether it is on the rise. Based on the search results, there were a total of 215 documents, of which 204 were articles and reviews. The statistical diagram of the number of annual publications and annual total publications showed that research in the field of gastric microbiota and gastric cancer first began in 1993. Since 2013, the annual number of publications in this field has shown a significant upward trend. The rapid growth rate from 2018 to 2022 indicated that research in this field has gradually gained more attention in recent years. In 2023, the annual number of publications decreased, which may be due to the fact that the search only ended on 18 July of that year (Figure 1A). The equation fitted according to the annual cumulative number of publications is *y* = 1.348 $e^{0.2847x}$ , *R*^2^ = 0.9818, which has good fitting properties and conforms to Price’s curve. Overall, the total number of publications and the annual publications in this field have grown exponentially.

#### 3.1.2 Analysis of funding

Analysis of the funding information for these publications shows that the cumulative number of funded publications has continued to increase since Web of Science began collecting funding information in 2008. It can be seen that the cumulative funding ratio has also increased steadily. From the analysis of annual funding, we can see that the annual funding rate for publications in this field has been rising steadily since 2013. This shows the attention and investment of researchers, indicating that this field has good development prospects (Figure 1A). According to our analysis, the top eight productive countries were selected for funding analysis. The stacked area chart of annually funded publications shows that in the early years, countries such as the United States and Korea had more funds invested in this field, while since 2021, China has had the largest proportion of funds invested in the field and the greatest output, indicating that China attaches more importance to research in the fields of gastric microbiota and gastric cancer (Figure 1B).

### 3.2 Researchers’ analysis

#### 3.2.1 Country analysis

**Figure 2:**
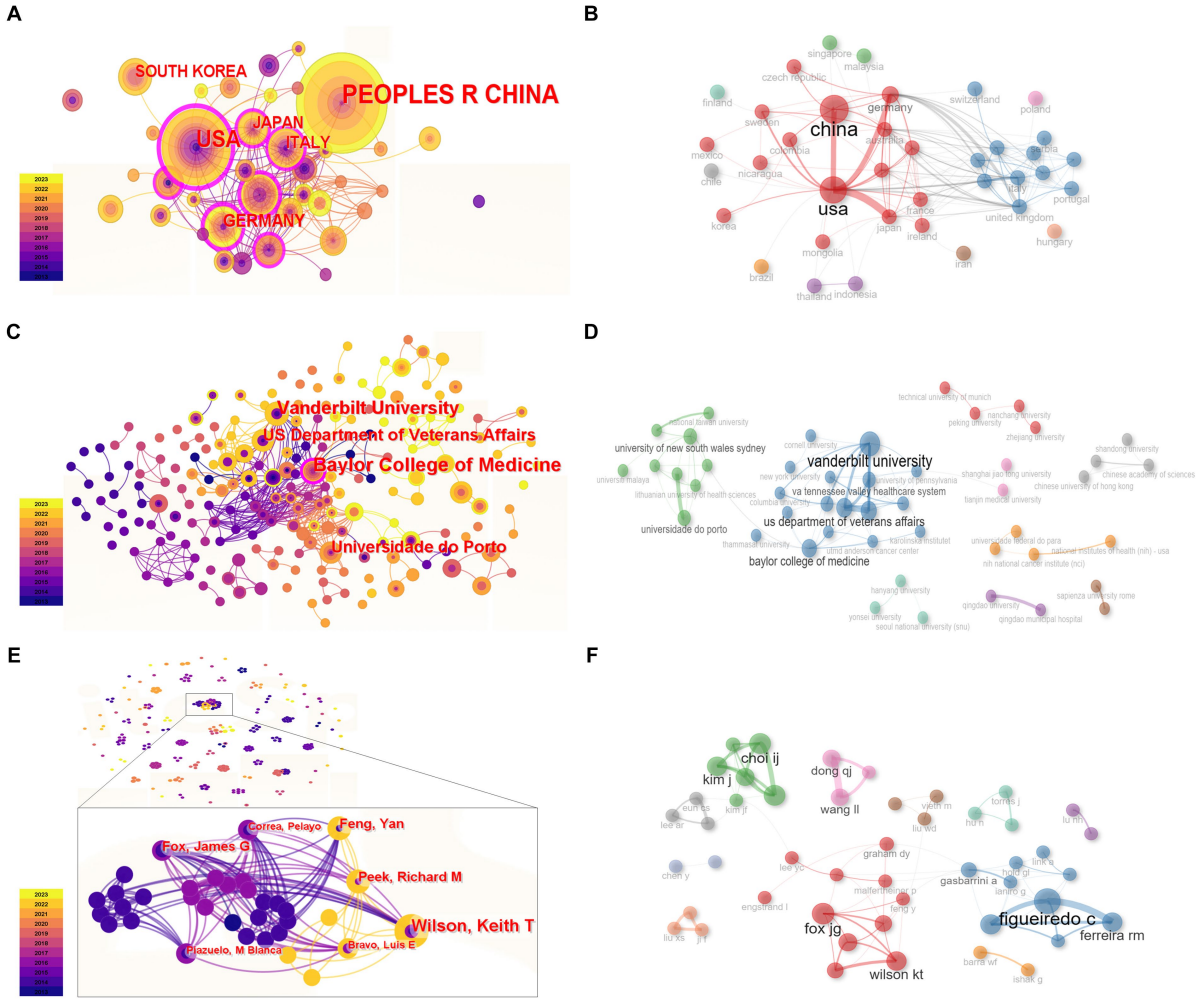
The co-occurrence and cooperation network of researchers. **(A,B)** Countries. **(C,D)** Institutions. **(E,F)** Authors. In panels **(A,C,E)**, each node was displayed as a growth ring. Nodes with betweenness centrality over 0.1 are shown by the purple outer ring.

The analysis of countries with relevant research from 2013 to 2023 showed that a total of 39 countries followed up on this field. Among them, China and the United States were the most productive countries (Table 1). In the co-occurrence graph, it can be seen that the United States started its research in this field earlier, and China has had an increasing number of prominent publications in recent years (Figure 2A). The betweenness centrality of the United States was the highest, reaching 0.43, which means the United States has a high influence in this field (Table 1). By analyzing the national cooperation network, it can be seen that China and the United States occupy a dominant position in the cooperation network. However, in general, the links between countries are thin and their colors are relatively dark, showing that the intensity of cooperation between countries in this field is low, and there has been poor cooperation in recent years (Figures 2A,B).

**Table 1:** The count of publications and the betweenness centrality of the top eight countries.

Rank	Count	Country	Rank	Centrality	Country
1	72	People’s Republic of China	1	0.43	United States
2	41	United States	2	0.29	Australia
3	15	Germany	3	0.23	Germany
4	14	Italy	4	0.15	Italy
5	13	Japan	5	0.15	United Kingdom
6	13	South Korea	6	0.14	Japan
7	11	Australia	7	0.11	Sweden
8	8	United Kingdom	8	0.1	Ireland
9	8	Portugal	9	0.06	Greece

#### 3.2.2 Institution analysis

An analysis of institutions showed that there was no prominent institution with a high publication volume. Among all the institutions, Baylor College of Medicine has published the most papers and has the highest betweenness centrality at 0.1. Except for Baylor College of Medicine, there is no institution with a centrality over 0.1, which means the bridge effect of each institution and the cooperation network are weak (Table 2). This might be due to the number of institutions being too high, while the difference in co-occurrence frequency is small. The institution cooperation network analysis showed that Vanderbilt University, the US Department of Medicine, Baylor College of Medicine, Universidade do Porto, etc. have cooperated to a certain extent (Figures 2C,D).

**Table 2:** The count of publications and the betweenness centrality of the top 12 institutions.

	Count	Centrality	Institution
1	9	0.1	Baylor College of Medicine
2	8	0.03	Vanderbilt University
3	7	0	Nanchang University
4	6	0	US Department of Veterans Affairs
5	6	0	Zhejiang University
6	6	0.01	Universidade do Porto
7	5	0	Chinese Academy of Sciences
8	5	0	Hanyang University
9	5	0	University of New South Wales Sydney
10	5	0	Veterans’ Health Administration (VHA)
11	5	0.02	Otto von Guericke University
12	5	0.03	Massachusetts Institute of Technology (MIT)

#### 3.2.3 Author analysis

Through statistics on the co-occurrence of the first author, it was found that the outputs of each single researcher in the field of gastric cancer and the gastric microbiota were even and relatively small, and the individual researchers exhibited a lack of centrality (Figure 2E). A major research group included Fox James G, Wilson Keith T, Feng Yan, Peek Richard M, etc. Researchers such as Figueiredo C and Ferreira RM, Kim J and Choi IJ, Dong QJ, and Wang LL also occupied a dominant position (Figures 2E,F). There was relatively less cooperation between authors. This result suggests that this field is in its infancy and has broad prospects. Co-citation network analysis of authors can reveal those who are under the spotlight and have made original contributions to a certain field. The CiteSpace analysis results show that Ferreira RM, Bik EM, and Coker OO had higher citation frequency, while Maldonado-Contreras A and Lofgren JL had high betweenness centrality (Table 3).

**Table 3:** The cited frequency and betweenness centrality of co-cited authors.

	Count	Year	Name		Centrality	Year	Name
1	97	2018	Ferreira RM	1	0.16	2013	Maldonado-Contreras A
2	92	2013	Bik EM	2	0.15	2013	Lofgren JL
3	91	2018	Coker OO	3	0.12	2014	Lertpiriyapong K
4	83	2013	Correa P	4	0.12	2016	Jo HJ
5	81	2015	Aviles-Jimenez F	5	0.11	2013	Correa P
6	80	2015	Eun CS	6	0.09	2017	Li TH
7	70	2013	Dicksved J	7	0.09	2014	Malfertheiner P
8	67	2013	Lofgren JL	8	0.08	2013	Bik EM
9	66	2014	Lertpiriyapong K	9	0.08	2013	Dicksved J
10	58	2016	Yang I	10	0.08	2014	El-Omar EM

### 3.3 Analysis of journals and documents

#### 3.3.1 Document analysis

**Figure 3:**
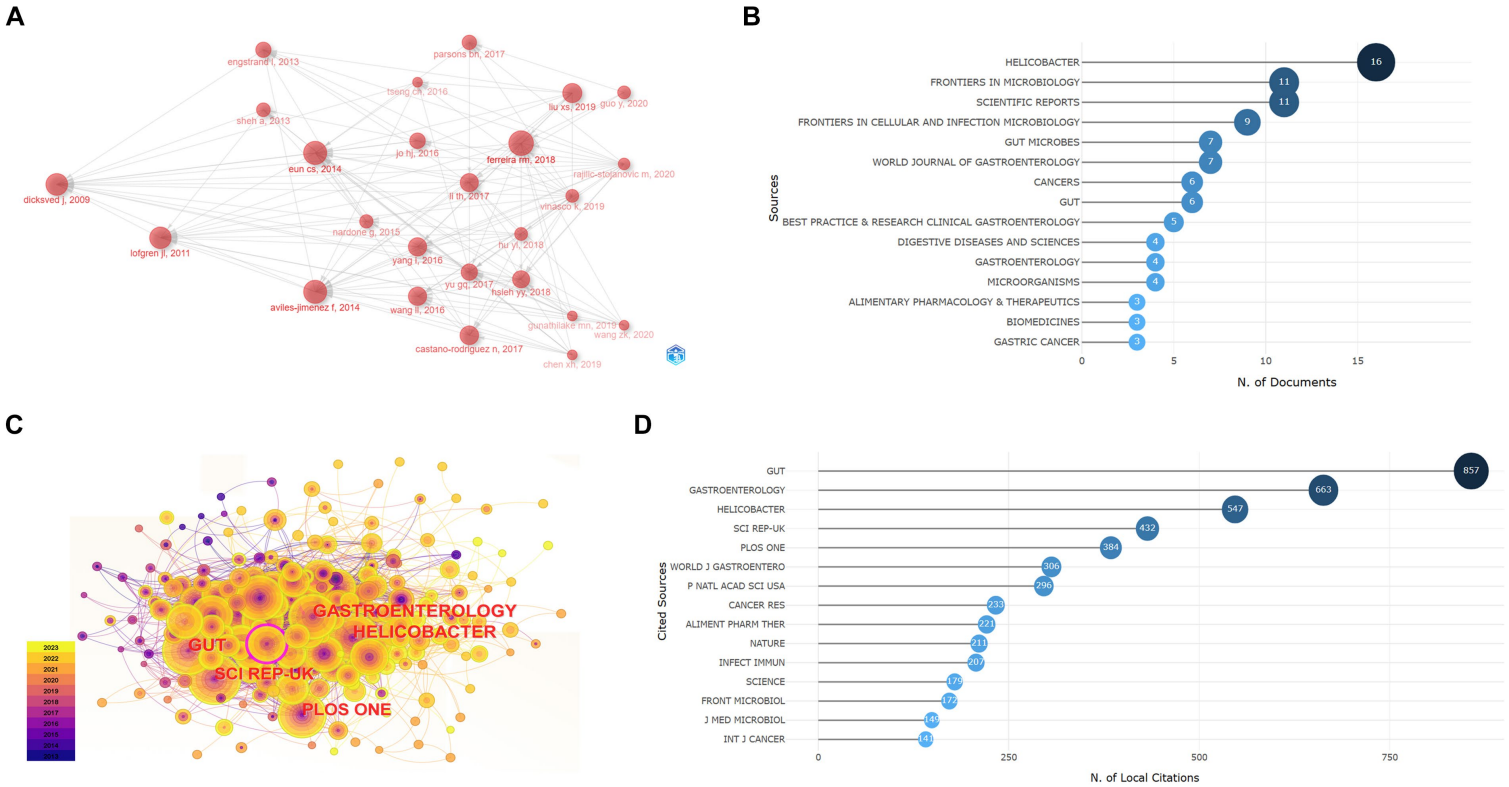
The documents and journal analysis. **(A)** Historiograph of citing documents. **(B)** Most relevant sources reflect the journal distribution of retrieval documents. **(C)** Co-citation network of reference journals. **(D)** Most locally cited sources show the source journal of references in the retrieval documents.

By analyzing citing documents, the latest research content and the research frontiers can be quickly found. LCS (local citation score) and GCS (global citation score) are two indicators of citing documents. After analyzing the publications retrieved from the WoSCC, it was found that the nodes representing studies by Ferreira RM, Eun CS, Aviles-Jimenez F, Lofgren JL, and Dicksved J were larger and had more pointed arrows, and these articles had the top five LCSs and GCSs, indicating that they were recognized by peers and researchers from other fields, which reflected the focus of this field to a certain extent (Figure 3A; Table 4).

**Table 4:** The citing documents with the top five LCS and GCS.

	Paper	Year	LCS		Paper	Year	GCS
1	Ferreira RM, 2018, Gut	2018	96	1	Ferreira RM, 2018, Gut	2018	338
	DOI: 10.1136/gutjnl-2017-314205				DOI: 10.1136/gutjnl-2017-314205		
2	Eun CS, 2014, Helicobacter	2014	82	2	Lofgren JL, 2011, Gastroenterology	2011	238
	DOI: 10.1111/hel.12145				DOI: 10.1053/j.gastro.2010.09.048		
3	Aviles-Jimenez F, 2014, Sci Rep-UK	2014	81	3	Dicksved J, 2009, J Med Microbiol	2009	214
	DOI: 10.1038/srep04202				DOI: 10.1099/jmm.0.007302-0		
4	Dicksved J, 2009, J Med Microbiol	2009	70	4	Aviles-Jimenez F, 2014, Sci Rep-UK	2014	205
	DOI: 10.1099/jmm.0.007302-0				DOI: 10.1038/srep04202		
5	Lofgren JL, 2011, Gastroenterology	2011	68	5	Eun CS, 2014, Helicobacter	2014	180
	DOI: 10.1053/j.gastro.2010.09.048				DOI: 10.1111/hel.12145		

LCS, local citation score; GCS, global citation score.

Document co-citation analysis refers to analyzing the references of retrieved documents to find documents cited by different documents at the same time, which helps to identify classic documents and reveal the knowledge basis of the research field. A co-citation analysis of references showed that documents published by Cocker OO and Ferreira RM in *Gut* in 2018 had the highest citation frequency, while an article by Lertpiriyapong K in *Gut* in 2014 had the highest betweenness centrality (Table 5).

**Table 5:** The cited frequency and betweenness centrality of co-cited publications.

	Count	Author and DOI		Centrality	Author and DOI
1	95	Ferreira RM, 2018, Gut	1	0.33	Lertpiriyapong K, 2014, Gut
		DOI: 10.1136/gutjnl-2017-314205			DOI: 10.1136/gutjnl-2013-305178
2	90	Coker OO, 2018, Gut	2	0.18	Coker OO, 2018, Gut
		DOI: 10.1136/gutjnl-2017-314281			DOI: 10.1136/gutjnl-2017-314281
3	50	Liu XS, 2019, Ebiomedicine	3	0.15	Lofgren JL, 2011, Gastroenterology
		DOI: 10.1016/j.ebiom.2018.12.034			DOI: 10.1053/j.gastro.2010.09.048
4	42	Li TH, 2017, Sci Rep-UK	4	0.14	Eun CS, 2014, Helicobacter
		DOI: 10.1038/srep44935			DOI: 10.1111/hel.12145
5	38	Hsieh YY, 2018, Sci Rep-UK	5	0.14	Li TH, 2017, Sci Rep-UK
		DOI: 10.1038/s41598-017-18596-0			DOI: 10.1038/srep44935
6	38	Castano-Rodriguez N, 2017, Sci Rep-UK	6	0.12	Yang I, 2016, Sci Rep-UK
		DOI: 10.1038/s41598-017-16289-2			DOI: 10.1038/srep18594
7	35	Yang I, 2016, Sci Rep-UK	7	0.11	Maldonado-Contreras A, 2011, ISME J
		DOI: 10.1038/srep18594			DOI: 10.1038/ismej.2010.149
8	35	Aviles-Jimenez F, 2014, Sci Rep-UK	8	0.11	Wang LL, 2016, Eur J Gastroen Hepat
		DOI: 10.1038/srep04202			DOI: 10.1097/MEG.0000000000000542
9	32	Lertpiriyapong K, 2014, Gut	9	0.11	Ferreira RM, 2018, Gut
		DOI: 10.1136/gutjnl-2013-305178			DOI: 10.1136/gutjnl-2017-314205
10	31	Eun CS, 2014, Helicobacter	10	0.1	Yu GQ, 2017, Front Cell Infect MI
		DOI: 10.1111/hel.12145			DOI: 10.3389/fcimb.2017.00302
11	31	Yu GQ, 2017, Front Cell Infect MI	11	0.09	Aviles-Jimenez F, 2014, Sci Rep-UK
		DOI: 10.3389/fcimb.2017.00302			DOI: 10.1038/srep04202
12	31	Schulz C, 2018, Gut	12	0.09	Liu XS, 2019, Ebiomedicine
		DOI: 10.1136/gutjnl-2016-312904			DOI: 10.1016/j.ebiom.2018.12.034

#### 3.3.2 Journal analysis

Relevant journal analysis reflects which journals are more interested in this field. Through our retrieval, the documents obtained were published in 109 journals and mainly distributed in the categories of Gastroenterology, Hepatology, Microbiology, and Oncology. The journal *Helicobacter* included the most articles in this field, reaching 16 (Figure 3B). The journal co-citation network shows that articles published in *Gut*, *Gastroenterology*, and *Helicobacter* had higher citation frequencies in this research field, and *Scientific Reports* had an important role with a centrality over 0.1 (Figure 3C). The analysis of the most locally cited sources showed the citation frequency of the source journals from which the references in our retrieval documents came. *Gut*, *Gastroenterology*, and *Helicobacter* had the highest citation frequency, which means they provided most of the research foundation (Figure 3D).

### 3.4 Keyword and research hotspot analysis

#### 3.4.1 Keyword frequency and co-occurrence analysis

**Figure 4:**
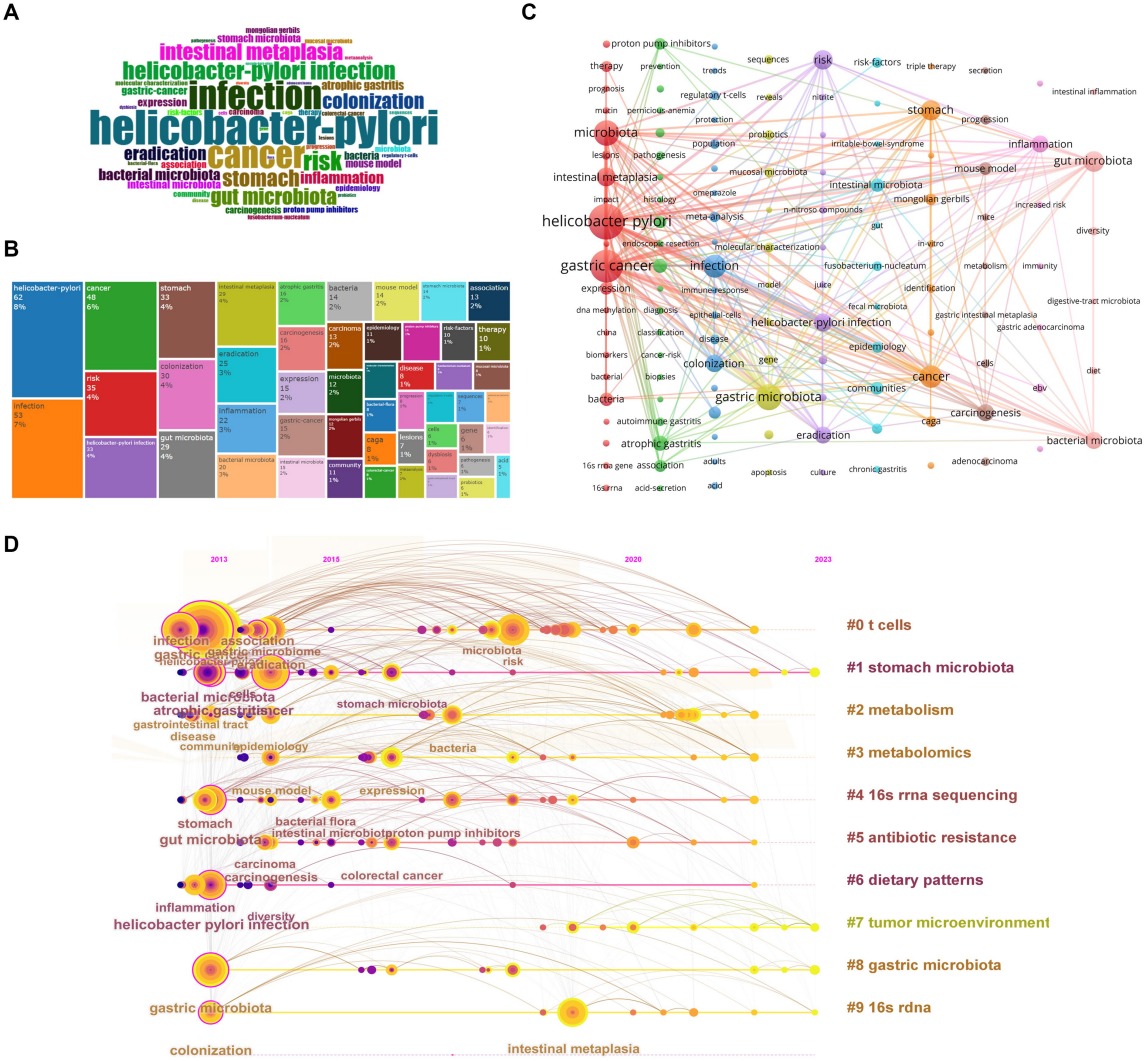
Keywords and cluster analysis. **(A)** Word cloud map. **(B)** Tree map of keyword frequency. **(C)** Cluster network of keywords produced by VOSviewer and Pajek. **(D)** Timeline cluster map of keywords.

Biblioshiny was used to conduct word frequency analysis of the top 50 keywords from 2013 to 2023. It can be seen that “*helicobacter-pylori*” appeared the most frequently. Other keywords with the highest word frequency included “infection”, “cancer”, “risk”, “gut microbiota”, “intestinal metaplasia”, “colonization”, “eradication”, “inflammation”, and so on (Figures 4A,B).

We also chose CiteSpace to analyze keywords from 2013 to 2023, with a total of 280 nodes and 1,557 links. Among the 280 keywords, the keywords with the highest frequency included “*Helicobacter pylori*”, “gastric cancer”, “infection”, etc., and the words with the highest betweenness centrality included “*Helicobacter pylori* infection”, “gut microbiota”, “atrophic gastritis”, etc. (Table 6). Our results show that in addition to search terms such as “gastric cancer” and “gastric microbiota”, there were keywords reflecting the precancerous conditions of GC, such as “atrophic gastritis”, “chronic gastritis”, “intestinal metaplasia”; keywords reflecting the eradication treatment of *H. pylori*, such as “*Helicobacter pylori*”, “eradication”, “proton pump inhibitors”, “antibiotics”, “clarithromycin”; keywords related to gastric microbiota research, such as “gut microbiota”, “colonization”, “*Fusobacterium nucleatum*”, “*Bifidobacterium*”, “mucosa associated microbiota”, “gastric non-*Helicobacter pylori* helicobacter”; keywords reflecting research technology, such as “16 s rRNA sequencing”, “next-generation sequencing”; and keywords reflecting pathogenic mechanisms, such as “inflammation”, “regulatory t cells”, “immune response”, “n nitroso compounds”, “DNA methylation”, “dendritic cells”, “kappa b activation”, “cdk12”, “CagA”, “e cadherin”, “ecl cell”, etc. (Supplementary Table 1).

**Table 6:** The frequency and betweenness centrality of the top 15 keywords.

	Count	Year	Keyword		Centrality	Year	Keyword
1	118	2013	*Helicobacter pylori*	1	0.18	2013	*Helicobacter pylori* infection
2	99	2013	Gastric cancer	2	0.17	2013	Gut microbiota
3	49	2013	Infection	3	0.15	2013	Atrophic gastritis
4	45	2014	Cancer	4	0.14	2014	Cancer
5	38	2013	Gastric microbiota	5	0.14	2013	Gastric microbiota
6	33	2013	Gut microbiota	6	0.13	2014	Association
7	33	2013	*Helicobacter pylori* infection	7	0.13	2013	Bacterial microbiota
8	31	2018	Risk	8	0.11	2013	Colonization
9	29	2019	Intestinal metaplasia	9	0.11	2013	Gastric cancer
10	28	2013	Colonization	10	0.11	2013	Infection
11	27	2013	Stomach	11	0.09	2019	Intestinal metaplasia
12	23	2014	Eradication	12	0.08	2013	Inflammation
13	22	2013	Atrophic gastritis	13	0.07	2014	Carcinoma
14	20	2013	Bacterial microbiota	14	0.07	2014	Eradication
15	18	2014	Gastric microbiome	15	0.06	2015	Digestive tract microbiota

#### 3.4.2 Keywords cluster analysis

Cluster analysis can be used to correlate keywords that appear at the same time in documents and cluster highly relevant words into categories so as to mine hidden information. Modularity Q (Q, value interval [0, 1]) and Weighted Mean Silhouette S (S, value interval [−1, 1]) are two important parameters of the clustering map. The Q-value can evaluate the quality of the clustering network. Q > 0.3 indicates that the network structure is persuasive. The S-value can measure the uniformity of cluster members. S > 0.5 indicates that the clustering results are reasonable.

We selected the log-likelihood ratio (LLR) algorithm to cluster 280 keywords from 2013 to 2023. The Q-value was 0.3971, and the S-value was 0.7278, indicating that the clustering results were informative and had reference significance. A total of 11 meaningful clusters were formed (Table 7). The smaller the cluster number, the more keywords were included. The keywords of each cluster partially overlap, which indicates that there is a correlation between the clusters. Through the artificial division of clusters, it can be seen that in the past 10 years, researchers in this field have mainly specialized in the mechanisms by which the gastric microbiota causes GC, including immunity (cluster #0), metabolism (clusters #2, #3), environmental factors (cluster #6), tumor microenvironment (cluster #7), and paying attention to the types of bacterial flora (clusters #1, #8) and research methods (clusters #4, #9). In addition, *H. pylori* eradication treatment (cluster #5, #10) has been one direction of research. Furthermore, *H. pylori* infection and its eradication treatment are closely related to changes in gastric flora richness and the occurrence of GC. Overall, these clusters are related to each other. In terms of research progress, the timeline map can reflect the time span of each cluster and the correlation between different clusters, reflecting the evolution of research. This showed that clusters #0, #1, #4, #6, and #8 already had important keywords in 2013. Among them, the keywords in clusters #0 and #1 had high frequency and wide relationships with other keywords. Cluster #7 had its first important keyword, “adverse prognosis”, in 2018, which indicates that these fields are just beginning to develop and need more complete and thorough research (Figure 4D).

**Table 7:** Keywords co-occurrence network clustering table.

Cluster ID	Size	Silhouette	Mean (year)	LLR
#0	56	0.61	2017	T cells (5.41, 0.05); gastrointestinal microbiota (5.41, 0.05); Barrett’s esophagus (5.41, 0.05); 16 s rRNA gene (4.23, 0.05); stomach cancer (4.17, 0.05)
T cells				
#1	36	0.59	2016	Stomach microbiota (16.2, 1.0E-4); bile acids (8.07, 0.005); bacterial microbiota (5.96, 0.05); accuracy rate (4.03, 0.05); identification (4.03, 0.05)
Stomach microbiota				
#2	33	0.707	2018	Metabolism (8.89, 0.005); disease (7.09, 0.01); epidemiology (5.3, 0.05); cytokines (5.3, 0.05); peptic ulcers (4.44, 0.05)
Metabolism				
#3	28	0.708	2018	Metabolomics (11.95, 0.001); pathogenesis (5.95, 0.05); distal gastric cancer (5.95, 0.05); association analysis (5.95, 0.05); thioredoxin (trxA; 5.95, 0.05)
Metabolomics				
#4	27	0.795	2016	16 s rRNA sequencing (12.9, 0.001); stomach (8.75, 0.005); s (8.59, 0.005); intestinal microbiota (8.59, 0.005); children (8.59, 0.005)
16 s rRNA sequencing				
#5	25	0.782	2017	Antibiotic resistance (10.91, 0.001); chronic gastritis (9.94, 0.005); carcinoma (7.22, 0.01); chronic intestinal inflammation (5.44, 0.05); alternative treatments (5.44, 0.05)
Antibiotic resistance				
#6	19	0.9	2014	Dietary patterns (5.03, 0.05); 16 s ribosomal RNA (5.03, 0.05); prevention (5.03, 0.05); altered Schaedler flora (5.03, 0.05); migrating motor complex (5.03, 0.05)
Dietary patterns				
#7	17	0.843	2021	Tumor microenvironment (10.55, 0.005); microbiota (microorganism; 7.95, 0.005); treatment (7.95, 0.005); host–microbe interactions (7.95, 0.005); TREGS (regulatory T cells; 7.95, 0.005)
Tumor microenvironment				
#8	16	0.893	2018	Gastric microbiota (11.05, 0.001); supplementation (5.37, 0.05); gastric non-*Helicobacter pylori* helicobacter (5.37, 0.05); Bifidobacterium (5.37, 0.05); prognosis (5.37, 0.05)
Gastric microbiota				
#9	14	0.754	2020	16 s rDNA (10.03, 0.005); animal models (5, 0.05); dysplasia (5, 0.05); *candida albicans* (5, 0.05); Epstein-Barr virus (5, 0.05)
16 s rDNA				
#10	6	0.963	2017	Containing triple therapy (9.17, 0.005); *Saccharomyces boulardii* supplementation (9.17, 0.005); containing quadruple therapy (9.17, 0.005); proton pump inhibitor (9.17, 0.005); low dose aspirin (9.17, 0.005)
Containing triple therapy				

LLR, log-likelihood ratio.

VOSviewer and Pajek were also used to generate clusters by analyzing keywords that appeared over 3 times. A total of 128 keywords were displayed, and 10 clusters were obtained (Figure 4C). Each color represents a thematic cluster with different numbers of keywords. The bigger the node, the greater the frequency of the keyword. Links between nodes signal the relationship between topics. The thicker the link, the greater the occurrence of co-occurrence between keywords. The red cluster contained keywords reflecting *H. pylori* infection in the process of gastric carcinogenesis and had tight links with other clusters through keywords such as “*Helicobacter pylori*”, “intestinal metaplasia”, and “expression.” The green cluster mainly focused on the diagnosis and treatment of patients with precancerous status but had few links with other clusters. The dark blue cluster reflected the treatment of *H. pylori* infection. The yellow cluster included keywords with respect to identifying different members of the gastric microbiota. The violet cluster consisted of keywords reflecting the pathogenic mechanism of *H. pylori*. The light blue cluster contained keywords for factors that belong to gastric microbiota members or influence their components. The orange cluster reflected the *in vitro* research techniques for *H. pylori* infection. Other clusters consisted of keywords reflecting research regarding other microbiota associated with the gastric microbiota. Among all the keywords, except for “gastric cancer” and “gastric microbiota”, other keywords making associations with other clusters were “*Helicobacter pylori*”, “infection”, “risk”, “intestinal metaplasia”, “inflammation”, “colonization”, and “eradication”, which reflected that the eradication of *H. pylori* and its pathogenic mechanisms got noticed. In addition, keywords such as “proton pump inhibitor”, “*Fusobacterium nucleatum*”, “autoimmune gastritis”, “atrophic gastritis”, “mucosal microbiota”, and “regulatory T cells” were closely related to those important keywords, which may represent new research directions.

#### 3.4.3 Research hotspot evolution

**Figure 5:**
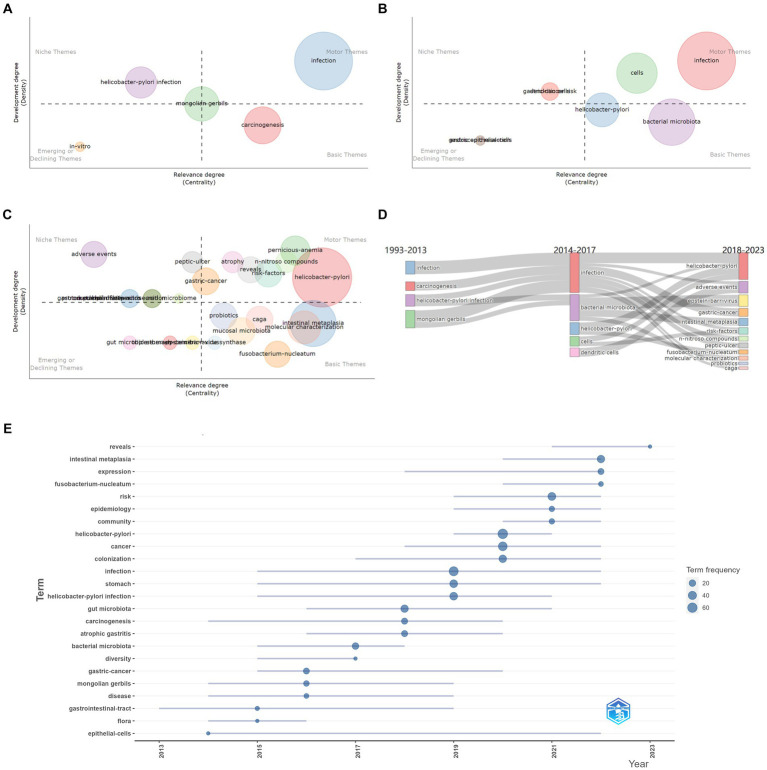
Research hotspot analysis. Panels **(A–C)** are the thematic maps of each time slice and describe the evolution trend of keywords. **(A)** represents the period of 1993–2013, **(B)** is for 2014–2017, and **(C)** is for 2018–2023. **(D)** Thematic evolution: the Sankey diagram shows the flow of research themes that merged or split. **(E)** Trend topic scatter diagram.

Keywords can reflect the core topics of research to some degree, helping to quickly elucidate the hotspots and progress in the research field. CiteSpace was used to undertake a burst analysis of keywords in the literature in this field from 2013 To 2023. A light blue line indicates that the keyword has not yet appeared, the dark blue line represents that the keyword has begun to appear, and the red line represents that the keyword has emerged (Table 8). Biblioshiny was applied to analyze the thematic evolution and trend topics. According to the distribution of publications per year, a Sankey diagram was drawn using the years 2013 and 2017 as the cutting points, and the thematic maps in each time slice were also exported (Figures 5A–D). In the thematic maps, each bubble represents an emerging topic that moves toward mainstream themes. The names of bubbles are keywords with the highest occurrence in the clusters. The bubble size is proportional to the word occurrences, and the position is determined by its centrality and density. in the Sankey diagram, each block represents a keyword, and its width represents its frequency. The width of links between blocks represents the strength of the association. By detecting the frequency of keywords plus in a certain period, a trend topic scatter diagram was drawn (Figure 5E). The horizontal axis displays the time when high-frequency keywords appear, and the vertical axis displays the first three topics for each year in decreasing order of frequency. Each bubble on the graph represents a topic. The reference year for each topic is identified using the median of the distribution of occurrence over the time period considered, while the bar indicates the first and third quartiles of the occurrence distribution.

**Table 8:** Emergent analysis of the top 64 keywords with the strongest citation bursts.

Keywords	Year	Strength	Begin	End	2013–2023
Bacterial microbiota	2013	4.47	2013	2017	▃▃▃▃▃▂▂▂▂▂▂
Disease	2013	1.53	2013	2014	▃▃▂▂▂▂▂▂▂▂▂
Gastric acid secretion	2013	1.49	2013	2016	▃▃▃▃▂▂▂▂▂▂▂
Atrophic gastritis	2013	1.4	2013	2013	▃▂▂▂▂▂▂▂▂▂▂
Endoscopic resection	2013	1.28	2013	2017	▃▃▃▃▃▂▂▂▂▂▂
Colitis	2013	1.2	2013	2014	▃▃▂▂▂▂▂▂▂▂▂
Flora	2014	2.12	2014	2016	▂▃▃▃▂▂▂▂▂▂▂
Cells	2014	2.08	2014	2017	▂▃▃▃▃▂▂▂▂▂▂
Diversity	2014	1.98	2014	2018	▂▃▃▃▃▃▂▂▂▂▂
Mouse model	2014	1.48	2014	2014	▂▃▂▂▂▂▂▂▂▂▂
Peptic ulcer	2014	1.27	2014	2014	▂▃▂▂▂▂▂▂▂▂▂
Molecular analysis	2014	1.27	2014	2014	▂▃▂▂▂▂▂▂▂▂▂
Dendritic cells	2014	1.16	2014	2015	▂▃▃▂▂▂▂▂▂▂▂
Immune response	2014	1.14	2014	2014	▂▃▂▂▂▂▂▂▂▂▂
Epithelial cells	2014	1.14	2014	2014	▂▃▂▂▂▂▂▂▂▂▂
*Helicobacter pylori* infection	2013	1.13	2014	2015	▂▃▃▂▂▂▂▂▂▂▂
Digestive tract microbiota	2015	1.57	2015	2017	▂▂▃▃▃▂▂▂▂▂▂
*H. pylori*	2015	1.37	2015	2018	▂▂▃▃▃▃▂▂▂▂▂
Stomach microbiota	2016	2.87	2016	2017	▂▂▂▃▃▂▂▂▂▂▂
Colorectal cancer	2016	1.63	2016	2018	▂▂▂▃▃▃▂▂▂▂▂
Mongolian gerbils	2016	1.5	2016	2019	▂▂▂▃▃▃▃▂▂▂▂
Identification	2016	1.29	2016	2016	▂▂▂▃▂▂▂▂▂▂▂
Mice	2016	1.29	2016	2016	▂▂▂▃▂▂▂▂▂▂▂
Cancer risk	2017	1.32	2017	2020	▂▂▂▂▃▃▃▃▂▂▂
China	2017	1.27	2017	2019	▂▂▂▂▃▃▃▂▂▂▂
Pathology	2017	1.25	2017	2017	▂▂▂▂▃▂▂▂▂▂▂
*H. pylori*	2017	1.25	2017	2017	▂▂▂▂▃▂▂▂▂▂▂
Kegg modules	2017	1.25	2017	2017	▂▂▂▂▃▂▂▂▂▂▂
Gastric cancer risk	2017	1.25	2017	2017	▂▂▂▂▃▂▂▂▂▂▂
Proton pump inhibitors	2017	1.21	2017	2020	▂▂▂▂▃▃▃▃▂▂▂
Intestinal microbiota	2015	1.13	2017	2017	▂▂▂▂▃▂▂▂▂▂▂
Association	2014	2.27	2018	2019	▂▂▂▂▂▃▃▂▂▂▂
Eradication	2014	2.24	2018	2020	▂▂▂▂▂▃▃▃▂▂▂
Risk	2018	1.74	2018	2023	▂▂▂▂▂▃▃▃▃▃▃
Meta-analysis	2018	1.58	2018	2019	▂▂▂▂▂▃▃▂▂▂▂
Autoimmune gastritis	2018	1.3	2018	2020	▂▂▂▂▂▃▃▃▂▂▂
Pernicious anemia	2018	1.26	2018	2018	▂▂▂▂▂▃▂▂▂▂▂
Trends	2018	1.26	2018	2018	▂▂▂▂▂▃▂▂▂▂▂
Adults	2018	1.2	2018	2018	▂▂▂▂▂▃▂▂▂▂▂
Progression	2019	2.79	2019	2020	▂▂▂▂▂▂▃▃▂▂▂
Molecular characterization	2019	2.18	2019	2021	▂▂▂▂▂▂▃▃▃▂▂
Diagnosis	2019	1.85	2019	2020	▂▂▂▂▂▂▃▃▂▂▂
Tumor microenvironment	2019	1.41	2019	2019	▂▂▂▂▂▂▃▂▂▂▂
Sequences	2020	1.79	2020	2021	▂▂▂▂▂▂▂▃▃▂▂
Juice	2020	1.2	2020	2020	▂▂▂▂▂▂▂▃▂▂▂
Next-generation sequencing	2020	1.2	2020	2020	▂▂▂▂▂▂▂▃▂▂▂
Nitrite	2020	1.2	2020	2020	▂▂▂▂▂▂▂▃▂▂▂
N nitroso compounds	2020	1.2	2020	2020	▂▂▂▂▂▂▂▃▂▂▂
Dysbiosis	2021	2.12	2021	2023	▂▂▂▂▂▂▂▂▃▃▃
Gastric microbiome	2014	1.87	2021	2023	▂▂▂▂▂▂▂▂▃▃▃
*Fusobacterium nucleatum*	2021	1.77	2021	2023	▂▂▂▂▂▂▂▂▃▃▃
Mucosa associated microbiota	2021	1.63	2021	2021	▂▂▂▂▂▂▂▂▃▂▂
Mucosal microbiota	2021	1.55	2021	2023	▂▂▂▂▂▂▂▂▃▃▃
Risk factors	2021	1.41	2021	2023	▂▂▂▂▂▂▂▂▃▃▃
Gastric carcinogenesis	2021	1.41	2021	2023	▂▂▂▂▂▂▂▂▃▃▃
Community	2013	1.12	2021	2023	▂▂▂▂▂▂▂▂▃▃▃
Intestinal metaplasia	2019	2.9	2022	2023	▂▂▂▂▂▂▂▂▂▃▃
Therapy	2019	1.97	2022	2023	▂▂▂▂▂▂▂▂▂▃▃
Inflammation	2013	1.85	2022	2023	▂▂▂▂▂▂▂▂▂▃▃
Expression	2016	1.57	2022	2023	▂▂▂▂▂▂▂▂▂▃▃
Gene	2018	1.35	2022	2023	▂▂▂▂▂▂▂▂▂▃▃
Apoptosis	2022	1.23	2022	2023	▂▂▂▂▂▂▂▂▂▃▃
Beta catenin	2022	1.23	2022	2023	▂▂▂▂▂▂▂▂▂▃▃
Adenocarcinoma	2016	1.14	2022	2023	▂▂▂▂▂▂▂▂▂▃▃

Light blue line: the year that keywords have not appeared; dark blue line: the year that keywords began to appear; red line: the year that keywords emerged.

In short, these knowledge mappings lead to the same conclusion. Early research focused on *H. pylori* infection and eradication as well as GC progression, and the research content was relatively simple, containing keywords such as “*Helicobacter pylori* infection”, “gastric acid secretion”, and “atrophic gastritis”, and “peptic ulcer”. the keyword “bacterial microbiota” had the highest burst strength and lasted for a long time, which reflected that the concept of the microbiota began to receive significant and sustained attention. At this time, the topic of this research field was homogeneous, and the frequency of each keyword was low.

In the mid-term, the topic was still limited, but the word frequency increased. Research regarding *H. pylori* infection was still at the center position. There were still some keywords indicating studies on *H. pylori* eradication regimen, such as “eradication” and “proton pump inhibitors”. The burst detection showed that researchers began to increase their research on the gastrointestinal microbiota, with keywords such as “gastric microbiome”, “mucosa associated microbiota”, “community”, and the strength of the keyword “stomach microbiota” reaching 2.87. There were also keywords reflecting related research methods such as “molecular characterization”, “kegg modules”, and “next-generation sequencing”. In this period, “bacterial microbiota” served as the basis of research. In addition, some new research topics appeared, for example, “dendritic cells”, which reflected the focus on the mechanisms of gastric carcinogenesis caused by the gastric microbiota. The pathogenic mechanisms of the gastric microbiota in GC mainly included immune and “inflammation” mechanisms, reflected in keywords such as “dendritic cells”, “immune response”, and “inflammation”.

In recent years, the research topics have become heterogeneous, which is evidenced by the increasing number of topic words with high Callon centrality. In combination with burst detection, thematic evolution, and hotspot trend analysis, it can be inferred that researchers have paid more attention to the richness of bacterial flora and the mechanisms by which different microorganisms cause GC during this stage. Specifically, topics generated included those reflecting the pathogenic mechanisms of *H. pylori*, such as “*Helicobacter pylori*”, “n nitroso compounds” and “caga”, with burst keywords including “n nitroso compounds”, “nitrite”, “inflammation”, “apoptosis”, “epithelial cells”, “beta catenin”; those reflecting different members of the gastric microbiome, such as “epstein barr virus”, “*Fusobacterium nucleatum*”, with burst keywords including “tumor microenvironment”, “mucosa associated microbiota”, “community”, “dysbiosis”, “*Fusobacterium nucleatum*”; those reflecting the safety of *H. pylori* eradication therapy, such as “adverse events”; and those reflecting the clinical treatment of the precancerous stage of GC, such as “peptic ulcer”, “intestinal metaplasia”, “risk factors”, and “probiotics”, with keywords such as “gastric carcinogenesis”. Notably, the burst keywords “dysbiosis”, “*Fusobacterium nucleatum*”, “mucosal microbiota”, “risk factors”, “gastric carcinogenesis”, “community”, “intestinal metaplasia”, “therapy”, “inflammation”, “expression”, “gene”, “apoptosis”, “beta catenin”, and “adenocarcinoma” are still in a burst period, leading researchers’ attention to changes in the gastric microbiota and its clinical application, and the pathogenic mechanisms of *H. pylori* and other non-*helicobacter* bacteria. Among them, the thematic map of the time slice 2018 to 2023 suggested that topics such as “*Fusobacterium nucleatum*”, “mucosal microbiota” and “intestinal metaplasia” in the lower right quadrant are basic and transversal themes and play an important role in the development of this field, but they were understudied and may therefore become hotspots in the future.

Generally speaking, research in this field at this stage is mainly divided into two aspects: the eradication and pathogenic mechanisms of *H. pylori*, which were well studied and becoming more important, and the role of different gastric microorganisms in the diagnosis and treatment of GC and their pathogenic mechanisms, which were less studied but important to this field. Among these topics, *H. pylori*, *Fusobacterium nucleatum*, immunity, and inflammation may be future research hotspots.

## 4 Discussion

This article is the first to identify the current research status, research hotspots, and trends in the field of gastric flora and gastric cancer based on bibliometric methods. The application of bibliometric analysis and visualization can display various topics and trends in basic or clinical research in order for researchers to carry out their work. In addition, our research shows that this research field has broad prospects and that there are still many clinical problems left to be solved.

While the annual cumulative number of papers has shown an exponential growth trend, in the past 3 years, the annual publications in this field have continued to grow rapidly, which suggests that the topic of gastric microbiota and GC has become popular. The analysis of funding showed that many countries are increasing capital input in this field, especially those that are productive. It can be predicted that in the future, the number of publications in this field will continue to grow exponentially, and funding support may also increase, making this field attractive for researchers.

Among the countries that published articles from 2013 to 2023, the total number of articles published by the top eight countries accounted for more than 90% of all articles published in this field, indicating that these countries were the main contributors to research in this field. Developed countries such as the United States and Germany published papers earlier, and their research results also had a relatively prominent influence. These countries often have advanced medical research institutions, outstanding scientific researchers, and sufficient financial support. As a developing country, China has been increasing its publication volume significantly since 2015, and the total number of articles quickly exceeded that of other countries. This may be due to the large population base, the large number of *H. pylori* infections and GC patients, and the popularization of endoscopic biopsy technology (Fan et al., 2021; Wang et al., 2022), as well as the rapid growth of national GDP and increased investment in scientific research (Lei et al., 2020; Zhou et al., 2021). However, although China is an important representative with respect to investment in this field, China’s betweenness centrality was not outstanding enough, indicating that Chinese researchers need to improve the quality of their research. The institutions that contributed the most to this field were mainly universities, mostly from the United States and European countries. Among them, Baylor College of Medicine has made prominent contributions and is considered one of the most outstanding medical schools in the United States. Through the analysis of authors and institutions, it can be seen that Fox JG and Wilson KT from Vanderbilt University, Figueiredo C and Ferreira RM from Universidade do Porto, have made great contributions in this field and published a series of highly influential articles, as well as actively participating in follow-up research. In recent years, with the largest number of publications, China’s output mainly came from Zhejiang University and Nanchang University. However, most researchers in this field currently tend to collaborate with domestic research institutions or within research groups in the same institution, even in productive countries. This might be due to the fact that this research field is only in its infancy. Therefore, as the number of publications increases, countries and institutions need to strengthen cooperation to promote the exchange of academic ideas and innovative development in the research field, which will also help to expand their influence.

In terms of journal analysis, the impact factor (IF) of a journal to some extent represents the influence of research in the field and the quality of the research results. The reference source journals have high IFs, indicating that the research foundation in this field is reliable. *Gut* was the most cited journal, indicating that the results published in this journal have an important influence in this research field, with an IF of 24.5. *Helicobacter* was the main journal publishing research in this field, with an IF of 4.4. Recently, journals such as *Frontiers in Microbiology*, *Scientific Reports*, and *Gut Microbes* have published numerous articles, among which the IF of *Gut Microbes* reached 12.2. However, compared to the cited literature, the current IFs of publications in this field are still lower in general. This may be due to the fact that research in this field has not attracted widespread attention, suggesting that researchers need to improve the quality of their research through reasonable experimental design and advanced research technology to produce more influential products and propose more novel perspectives.

A large number of studies have taken various directions, leading to pathogenesis research and clinical practical applications. By reading the key literature identified via the historiograph of citing documents, the research progression can be revealed. After identifying differences in the gastric microbiota characteristics between GC patients and those with dyspepsia, as the technique developed, the gradual shifting of the gastric microbiota in Correa’s cascade was confirmed, and genotoxic colonies were finally identified. In this process, the correlations between the gastric microbiota, gastric cancer, and specific pathogenic bacteria were gradually identified. The application of specific microbiota in the clinical identification of GC patients was gradually supported. Subsequently, through PICRUSt analysis and other means, the role of different gastric microorganisms in the progression of GC was gradually revealed. The article published by Ferreira RM et al. in *Gut* in 2018 (doi: 10.1136/gutjnl-2017-314205) had the highest citation frequency and the highest LCS and GCS, indicating that it was widely recognized by researchers as an important research basis. The researchers conducted a retrospective analysis of the gastric microbiota of patients with GC and chronic gastritis. They found that the diversity of the gastric microbiota was reduced in GC patients, and other bacterial genera, mainly intestinal commensal bacteria, were enriched, showing community characteristics different from chronic gastritis patients. This article revealed that gastric microbiota dysbiosis is related to GC and proved that the microbial dysbiosis index (MDI) can be used to identify GC, with the area under the curve (AUC) being 0.91 and 0.89, respectively (Ferreira et al., 2018). “Mucosal microbiome dysbiosis in gastric carcinogenesis,” published by Coker OO et al. (doi: 10.1136/gutjnl-2017-314281) close to the same period, also proved that there are differences in the gastric microbial composition and bacterial interactions during the progression from chronic gastritis to GC, and the correlation strengths between enriched groups and reduced groups increased (*p* < 0.001; Coker et al., 2018).

The visualization and clustering of keywords also reveal the evolution of the research topic. The timeline graph and thematic evolution analysis showed that although many important keywords such as “*Helicobacter pylori*” and “gastric cancer” have emerged in the early years, this research field is still developing and the topics described by these keywords are still being studied. In addition, topics that have begun to receive attention in recent years, such as “tumor microenvironment” and “stomach microbiota”, may be the focus of future research. In this research field, *H. pylori*, as a major member of the gastric microbiota, receives constant attention. Keyword analysis showed that “*helicobacter-pylori*”, “infection”, “eradication”, “proton pump inhibitors”, “regulatory T cells”, “CagA”, etc. were important keywords. As *H. pylori* is associated with GC and has a high infection rate in the general population, the diagnostic approaches, eradication methods, and carcinogenesis mechanisms for *H. pylori* have been extensively studied (Yang et al., 2014; Ansari and Yamaoka, 2022). However, current *H. pylori* eradication therapies face challenges involving antibiotic resistance (Ansari and Yamaoka, 2022; Suzuki et al., 2022), dysbiosis (Gotoda et al., 2018, 2020), the potential danger of long-term PPI use, and other shortcomings (Kuipers et al., 1996; Xu et al., 2013; Jiang et al., 2019; McCarthy, 2020; Seo et al., 2021; Arai et al., 2023). Recent studies have shown that *H. pylori* eradication regimens based on Vonoprazan (VPZ) are effective and safe (Kakiuchi et al., 2023) and have a higher *H. pylori* eradication rate than PPI-containing triple or quadruple therapy (Chey et al., 2022). The dual therapy consisting of VPZ and amoxicillin has a low incidence of adverse reactions and can also avoid unnecessary use of antibiotics, reducing the incidence of dysbiosis of the intestinal microbiota (Ouyang et al., 2022; Zhang J. et al., 2023). The dosage regimen of VPZ and the adverse events associated with its strong acid-suppressive effect still need further evaluation (Hu et al., 2022; Arai et al., 2023). In addition, the eradication of *H. pylori* might not be necessary for children because infection by *H. pylori* has a protective effect against asthma and inflammatory bowel disease via the systemic immune tolerance induced by dendritic cells (DCs) and regulatory T cells (Treg cells; Ravikumara, 2023). *Helicobacter pylori* infection or seropositivity have a detrimental impact on the efficacy of cancer immunotherapies, and the eradication of *H. pylori* infection by antibiotherapy does not revert the *H. pylori*-induced hyporesponsiveness to cancer immunotherapy (Che et al., 2022; Oster et al., 2022a,b; Gong et al., 2023). However, another meta-analysis has suggested that GC patients with *H. pylori* infection may respond better to PD-1/PD-L1 blockade therapy (Zhu et al., 2023). These divergent findings may be explained by differences in patients and treatment characteristics, as well as potential confounding factors. Thus, many experts are calling for a more individualized eradication approach in the context of additional risk factors rather than unconditionally eradicating *H. pylori* in every case. Clarifying the pathogenesis of *H. pylori* is still an important task. *Helicobacter pylori* infection triggers complex chronic immune responses, leading to the occurrence of a variety of diseases. *Helicobacter pylori* virulence factors such as cytotoxin-associated gene A (CagA), vacuolating cytotoxin A (VacA), and *Helicobacter pylori* neutrophil-activating protein (HP-NAP) significantly affect the function of DCs in tumor microenvironment or bone marrow-derived DCs and play an important role in the induction of GC (Fu and Lai, 2023; Raspe et al., 2023; Yuan et al., 2023). The Treg cell-mediated inflammatory response caused by *H. pylori* infection (Bagheri et al., 2016, 2018; Owyang et al., 2020) and the genome instability caused by CagA (Bagheri et al., 2018; Takahashi-Kanemitsu et al., 2020; Alipour, 2021; Imai et al., 2021; Murata-Kamiya and Hatakeyama, 2022; Marshall, 2023) have been extensively studied. Recent research has shown that *H. pylori* can inhibit miRNA-375 expression in the stomach. Downregulated miR-375 activates the JAK2-STAT3 pathway, which then promotes the secretion of IL-6, IL-10, and VEGF, leading to the immature differentiation of DCs and the induction of GC (Zhang et al., 2021). Notch signaling regulates the function and phenotype of DCs, thus mediating the differentiation of CD4^+^ T cells during *H. pylori* infection (Liu et al., 2023).

As 16 s rRNA sequencing techniques developed and gut microbiome research boomed with the confirmation of the relationship between the gastric microbiota and gastric carcinogenesis, researchers began to turn their attention to the study of the gastric microbiota. In this respect, “gut microbiota”, “colonization”, “16 s rRNA sequencing”, “risk”, “atrophic gastritis”, and “intestinal metaplasia” are important keywords, indicating that the clinical application of gastric microbiota in GC risk assessment and treatment are also current hotspots. As *H. pylori* is a bacterium that affects and is affected by the gastric microbiota, it is closely related to other bacteria in the progression of GC. Non-*H. pylori* microorganisms interact with *H. pylori* in gastric carcinogenesis (Guo et al., 2023). Successful *H. pylori* eradication can reverse gastric microbiota dysbiosis (Guo et al., 2020; Guo Q. et al., 2022), and its high eradication rate is related to specific flora members (Niu et al., 2021). The intestinal microbiota of *H. pylori*-positive GC patients is also transformed, and this may further contribute to GC (Gao et al., 2018; Dash et al., 2019; Seol et al., 2019; Iino and Shimoyama, 2021). Modulation of the gastrointestinal microbiota is beneficial to the eradication of *H. pylori* and the treatment of gastric diseases related to microbial dysbiosis (Viazis et al., 2022; Musazadeh et al., 2023; Zhang L. et al., 2023). The study of specific strains or pathogenic pathways of non-*H. pylori* bacteria in GC progression is helpful in identifying relevant treatment measures accordingly. Using 16 s rRNA sequencing technology to analyze gastric epithelial bacteria at different stages of GC progression, it was found that some bacterial taxa, such as *Peptostreptococcus stomatis*, *Streptococcus anginosus*, *Parvimonas micra*, etc., were significantly enriched in GC patients and could be used to identify precancerous lesions and GC (Coker et al., 2018; Liu et al., 2022). Microbial taxonomic features (MTFs) can be used to predict early gastric neoplasia (EGN; Png et al., 2022) and may improve the accuracy of the polygenic risk score (PRS) model in predicting GC (Wang et al., 2023). As for the mechanism, many studies have revealed that non-*H. pylori* microorganisms promote GC by inducing inflammation, modulating the immune response, triggering DNA damage, and promoting epithelial–mesenchymal transformation (Yang et al., 2022; Liao et al., 2023). Gastric non-*H. pylori* microorganisms may participate in the progression of GC by affecting host DNA methylation (Yue et al., 2023). Different bacterial taxa are related to certain types of infiltrating immune cells (Liao et al., 2023). In the gastric microbiota associated with atrophy/intestinal metaplasia, functional pathways such as amino acid metabolism and inositol phosphate metabolism are enriched, while folate biosynthesis and NOD-like receptor signaling are reduced, which may explain the ongoing progression of precancerous conditions even after *H. pylori* eradication (Sung et al., 2020). Research on the intestinal microbiota continuously activating host immunity and producing a variety of metabolites has illuminated its effect on GC (Nasr et al., 2020; Guo Y. et al., 2022), while the GC microflora can modulate macrophages and enhance gastric tumor development by suppressing antitumor immunity, activating oncogenic signaling pathways, and producing protumor metabolites (Zhang W. et al., 2023).

Through emergent analysis, we can speculate that the role of *Fusobacterium nucleatum* (*F. nucleatum*) in the Correa cascade of GC development may become a research hotspot in the future. *Fusobacterium nucleatum*, which exists in the oral cavity and gastrointestinal tract of humans, is an opportunistic pathogen causing systematic diseases, for example, gastrointestinal cancers (Chen et al., 2022; He Z. et al., 2022). A number of studies have demonstrated the potential pathogenic role of *F. nucleatum* in colorectal cancer (CRC; Lee et al., 2019). *Fusobacterium nucleatum* causes CRC by adhering and forming biofilm, invading host cells, producing metabolites, and releasing vesicles (Chen et al., 2022). *Fusobacterium nucleatum* promotes CRC metastasis through M2 polarization of macrophages in the tumor microenvironment (Chen et al., 2018; Xu et al., 2021). However, its roles in GC are not so clear. In a study using *Clostridium* and *Fusobacterium nucleatum* in biopsy tissue to diagnose GC, the sensitivity reached 100%, the specificity was 68.8%, and the AUC was 0.875 (Hsieh et al., 2018). The combined colonization of *F. nucleatum* and *Helicobacter pylori* has also been associated with a poor survival rate in late-stage GC patients treated with gastrectomy, suggesting that it may promote the progression or metastasis of GC by synergizing with *H. pylori* (Hsieh et al., 2021, 2023). *Fusobacterium nucleatum* has strong interactions with *Porphyromonas*, *Prevotella*, etc., which may lead to shortened survival (Nie et al., 2021; Lehr et al., 2023). A bioinformatic analysis suggested that neutrophil transcriptional activation induced by *F. nucleatum* may be implicated in the occurrence of GC through several candidate genes, including DNAJB1, EHD1, IER2, CANX, and PH4B. Functional analysis showed that membrane-bound organelle dysfunction, intracellular trafficking, transcription factors ER71 and Sp1, and miR580 and miR155 were other candidate mechanisms (Zhou et al., 2022). The metabolic function analysis showed that *F. nucleatum*-positive GC tissues were significantly enriched in the biosynthesis of lysine, peptidoglycan, and tRNA metabolic functions (Nie et al., 2021). These results await further verification. Existing studies have demonstrated the role of *F. nucleatum* in the ERBB2-PIK3-AKT–mTOR pathway and the miR-885-3p/EphB2/PI3K/AKT axis (Hsieh et al., 2023; Xin et al., 2023). However, some researchers have questioned the actual role of *F. nucleatum* in gastric carcinogenesis. An *F. nucleatum*-positive result was associated with poor prognosis in patients with Lauren’s diffuse type GC but had no association with the prognosis of intestinal-type GC. These results still remain to be confirmed (Boehm et al., 2020; Nie et al., 2021). *Fusobacterium nucleatum* may promote carcinogenesis via *Fusobacterium* adhesionA (FadA), which binds to E-cadherin, activating Wnt/β-catenin signaling and various inflammatory and oncogenic properties of the cells (Rubinstein et al., 2013, 2019). Since the diffuse type of GC is strongly associated with E-cadherin deregulation, one may speculate on the potential molecular mimicry and specific prognostic relevance of *F. nucleatum* to the diffuse type of GC (Boehm et al., 2020). Furthermore, the interaction of *F. nucleatum* with non-*H. pylori* gastric microorganisms also requires more explanation.

This study has some limitations. Only relevant studies published by WoSCC were included in this study; cutting-edge research with high quality from other databases such as PubMed and Scopus might have been ignored. Second, the language was restricted to English, which may have excluded high-quality literature in other languages. In addition, the co-citation frequency of the literature is time-dependent, and since the research in this field is still in the developing stage, the number of citations cannot accurately reflect the importance of the document, especially important documents published in recent years. With the exponential growth of publications in this field, our research needs to be constantly updated to keep up with the latest research developments. Finally, due to missing keywords reported by Biblioshiny in some literature, the outcome of the CiteSpace keyword analysis might be a little inaccurate; however, we used different bibliometric software to conduct our analysis and verify the results.

## 5 Conclusion

To the best of our knowledge, this study is the first to use visualization software and data mining methods to conduct a bibliometric analysis of publications in the field of gastric microbiota and gastric cancer and to determine the research status, hotspots, and development trends in this field. Research in this field has mainly focused on the eradication therapy and pathogenic mechanisms of *Helicobacter pylori*, as well as the utilization of gastric microbiota in the evaluation and treatment of gastric cancer. Future research hotspots may include the use of the gastric microorganisms represented by *Fusobacterium nucleatum* in the diagnosis of and for therapeutic effects on gastric cancer. Their mechanisms of action need to be further explored in order to provide a theoretical basis for clinical application. Relevant researchers or researchers outside this field can use this study to improve their awareness and understanding of the field and to gain some perspectives for further research.

## Data availability statement

All data used for our research is available through our retrieval query. Further inquiries can be directed to the corresponding author.

## Author contributions

YK: Data curation, Formal analysis, Investigation, Visualization, Writing – original draft. CT: Data curation, Formal analysis, Investigation, Visualization, Writing – original draft. JZ: Formal analysis, Investigation, Methodology, Writing – review & editing. WD: Conceptualization, Methodology, Writing – review & editing.

## References

[ref1] Alipour M. (2021). Molecular mechanism Of*helicobacter pylori*-induced gastric Cancer. J. Gastrointest. Cancer 52, 23–30. doi: 10.1007/s12029-020-00518-5, PMID: 32926335 PMC7487264

[ref2] Ansari S. Yamaoka Y. (2022). *Helicobacter pylori* infection, its laboratory diagnosis, and antimicrobial resistance: a perspective of clinical relevance. Clin. Microbiol. Rev. 35:e0025821. doi: 10.1128/cmr.00258-21, PMID: 35404105 PMC9491184

[ref3] Arai J. Hayakawa Y. Niikura R. Ihara S. Aoki T. Honda T. . (2023). Letter: potassium-competitive acid blockers may increase the risk of gastric cancer after *Helicobacter pylori* eradication a retrospective multicentre-cohort analysis. Aliment. Pharmacol. Ther. 57, 1196–1198. doi: 10.1111/apt.17461, PMID: 37094303

[ref4] Aria M. Cuccurullo C. (2017). *Bibliometrix*: an R-tool for comprehensive science mapping analysis. J. Informet. 11, 959–975. doi: 10.1016/j.joi.2017.08.007

[ref5] Arnold M. Park J. Y. Camargo M. C. Lunet N. Forman D. Soerjomataram I. (2020). Is gastric cancer becoming a rare disease? A global assessment of predicted incidence trends to 2035. Gut 69, 823–829. doi: 10.1136/gutjnl-2019-320234, PMID: 32001553 PMC8520492

[ref6] Aviles-Jimenez F. Vazquez-Jimenez F. Medrano-Guzman R. Mantilla A. Torres J. (2014). Stomach microbiota composition varies between patients with non-atrophic gastritis and patients with intestinal type of gastric cancer. Sci. Rep. 4:4202. doi: 10.1038/srep04202, PMID: 24569566 PMC3935187

[ref7] Bagheri N. Azadegan-Dehkordi F. Rahimian G. Rafieian-Kopaei M. Shirzad H. (2016). Role of regulatory T-cells in different clinical expressions of *Helicobacter pylori* infection. Arch. Med. Res. 47, 245–254. doi: 10.1016/j.arcmed.2016.07.013, PMID: 27664483

[ref8] Bagheri N. Salimzadeh L. Shirzad H. (2018). The role of T helper 1-cell response in *Helicobacter pylori*-infection. Microb. Pathog. 123, 1–8. doi: 10.1016/j.micpath.2018.06.033, PMID: 29936093

[ref9] Bik E. M. Eckburg P. B. Gill S. R. Nelson K. E. Purdom E. A. Francois F. . (2006). Molecular analysis of the bacterial microbiota in the human stomach. Proc. Natl. Acad. Sci. U. S. A. 103, 732–737. doi: 10.1073/pnas.0506655103, PMID: 16407106 PMC1334644

[ref10] Boehm E. T. Thon C. Kupcinskas J. Steponaitiene R. Skieceviciene J. Canbay A. . (2020). *Fusobacterium nucleatum* is associated with worse prognosis in Lauren's diffuse type gastric cancer patients. Sci. Rep. 10:16240. doi: 10.1038/s41598-020-73448-8, PMID: 33004953 PMC7530997

[ref11] Castano-Rodriguez N. Goh K. Fock K. M. Mitchell H. M. Kaakoush N. O. (2017). Dysbiosis of the microbiome in gastric carcinogenesis. Sci. Rep. 7:15957. doi: 10.1038/s41598-017-16289-2, PMID: 29162924 PMC5698432

[ref12] Che H. Xiong Q. Ma J. Chen S. Wu H. Xu H. . (2022). Association of *Helicobacter pylori* infection with survival outcomes in advanced gastric cancer patients treated with immune checkpoint inhibitors. BMC Cancer 22:904. doi: 10.1186/s12885-022-10004-9, PMID: 35986342 PMC9389789

[ref13] Chen C. M. (2006). CiteSpace II: detecting and visualizing emerging trends and transient patterns in scientific literature. J. Am. Soc. Inf. Sci. Technol. 57, 359–377. doi: 10.1002/asi.20317

[ref14] Chen C. Hu Z. Liu S. Tseng H. (2012). Emerging trends in regenerative medicine: a scientometric analysis in CiteSpace. Expert Opin. Biol. Ther. 12, 593–608. doi: 10.1517/14712598.2012.674507, PMID: 22443895

[ref15] Chen Y. Huang Z. Tang Z. Huang Y. Huang M. Liu H. . (2022). More than just a periodontal pathogen -the research Progress on *Fusobacterium nucleatum*. Front. Cell. Infect. Microbiol. 12:5318. doi: 10.3389/fcimb.2022.815318, PMID: 35186795 PMC8851061

[ref16] Chen T. Li Q. Wu J. Wu Y. Peng W. Li H. . (2018). *Fusobacterium nucleatum* promotes M2 polarization of macrophages in the microenvironment of colorectal tumours via a TLR4-dependent mechanism. Cancer Immunol. Immunother. 67, 1635–1646. doi: 10.1007/s00262-018-2233-x, PMID: 30121899 PMC11028377

[ref17] Chen J. Nie S. Qiu X. Zheng S. Ni C. Yuan Y. . (2023). Leveraging existing 16S rRNA microbial data to identify diagnostic biomarker in Chinese patients with gastric cancer: a systematic meta-analysis. MSystems 8:e74723:e0074723. doi: 10.1128/msystems.00747-23, PMID: 37787561 PMC10654077

[ref18] Chey W. D. Megraud F. Laine L. Lopez L. J. Hunt B. J. Howden C. W. (2022). Vonoprazan triple and dual therapy for *Helicobacter pylori* infection in the United States and Europe: randomized clinical trial. Gastroenterology 163, 608–619. doi: 10.1053/j.gastro.2022.05.055, PMID: 35679950

[ref19] Cobo M. J. Lopez-Herrera A. G. Herrera-Viedma E. Herrera F. (2011). Science mapping software tools: review, analysis, and cooperative study among tools. J. Am. Soc. Inf. Sci. Technol. 62, 1382–1402. doi: 10.1002/asi.21525

[ref20] Coker O. O. Dai Z. Nie Y. Zhao G. Cao L. Nakatsu G. . (2018). Mucosal microbiome dysbiosis in gastric carcinogenesis. Gut 67, 1024–1032. doi: 10.1136/gutjnl-2017-314281, PMID: 28765474 PMC5969346

[ref21] Correa P. (1988). A human model of gastric carcinogenesis. Cancer Res. 48, 3554–3560. 3288329

[ref22] Correa P. (1992). Human gastric carcinogenesis: a multistep and multifactorial process--first American Cancer Society award lecture on Cancer epidemiology and prevention. Cancer Res. 52, 6735–6740. PMID: 1458460

[ref23] Dash N. R. Khoder G. Nada A. M. Al Bataineh M. T. (2019). Exploring the impact of *Helicobacter pylori* on gut microbiome composition. PLoS One 14:e0218274. doi: 10.1371/journal.pone.0218274, PMID: 31211818 PMC6581275

[ref24] Donthu N. Kumar S. Mukherjee D. Pandey N. Lim W. M. (2021). How to conduct a bibliometric analysis: an overview and guidelines. J. Bus. Res. 133, 285–296. doi: 10.1016/j.jbusres.2021.04.070

[ref25] Dracos A. Cognetti G. (1995). Scientific literature: bibliometric and bibliographic indicators as integrative criteria for an objective evaluation of research activity. Annali dell'Istituto Superiore Di Sanita 31, 381–390. PMID: 8712583

[ref26] Esfahani H. Tavasoli K. Jabbarzadeh A. (2019). Big data and social media: a scientometrics analysis. Int J Data Netw Sci 3, 145–164. doi: 10.5267/j.ijdns.2019.2.007

[ref27] Fan X. Qin X. Zhang Y. Li Z. Zhou T. Zhang J. . (2021). Screening for gastric cancer in China: advances, challenges and visions. Chin. J. Cancer Res. 33, 168–180. doi: 10.21147/j.issn.1000-9604.2021.02.05, PMID: 34158737 PMC8181866

[ref28] Ferreira R. M. Pereira-Marques J. Pinto-Ribeiro I. Costa J. L. Carneiro F. Machado J. C. . (2018). Gastric microbial community profiling reveals a dysbiotic cancer-associated microbiota. Gut 67, 226–236. doi: 10.1136/gutjnl-2017-314205, PMID: 29102920 PMC5868293

[ref29] Fu H. Lai Y. (2023). The role of *Helicobacter pylori* neutrophil-activating protein in the pathogenesis of *H. Pylori* and beyond: from a virulence factor to therapeutic targets and therapeutic agents. Int. J. Mol. Sci. 24:91. doi: 10.3390/ijms24010091, PMID: 36613542 PMC9820732

[ref30] Gao J. Zhang Y. Gerhard M. Mejias-Luque R. Zhang L. Vieth M. . (2018). Association between gut microbiota and *Helicobacter pylori*-related gastric lesions in a high-risk population of gastric Cancer. Front. Cell. Infect. Microbiol. 8:202. doi: 10.3389/fcimb.2018.00202, PMID: 29971220 PMC6018392

[ref31] Gong X. Shen L. Xie J. Liu D. Xie Y. Liu D. (2023). *Helicobacter pylori* infection reduces the efficacy of cancer immunotherapy: a systematic review and meta-analysis. Helicobacter 28:e13011. doi: 10.1111/hel.13011, PMID: 37661590

[ref32] Gotoda T. Kusano C. Suzuki S. Horii T. Ichijima R. Ikehara H. (2020). Clinical impact of vonoprazan-based dual therapy with amoxicillin For*h. pylori*infection in a treatment-naive cohort of junior high school students in Japan. J. Gastroenterol. 55, 969–976. doi: 10.1007/s00535-020-01709-4, PMID: 32666199

[ref33] Gotoda T. Takano C. Kusano C. Suzuki S. Ikehara H. Hayakawa S. . (2018). Gut microbiome can be restored without adverse events after *Helicobacter pylori* eradication therapy in teenagers. Helicobacter 23:e12541. doi: 10.1111/hel.12541, PMID: 30311721

[ref34] Guo Y. Cao X. S. Guo G. Y. Zhou M. G. Y. (2022). Effect of *Helicobacter Pylori* eradication on human gastric microbiota: a systematic review and Meta-analysis. Front. Cell. Infect. Microbiol. 12:899248. doi: 10.3389/fcimb.2022.899248, PMID: 35601105 PMC9114356

[ref35] Guo Y. Cao X. Zhou M. Yu B. (2023). Gastric microbiota in gastric cancer: different roles of Helicobacter pylori and other microbes. Front. Cell. Infect. Microbiol. 12:5811. doi: 10.3389/fcimb.2022.1105811, PMID: 36704105 PMC9871904

[ref36] Guo Q. Qin H. Liu X. Zhang X. Chen Z. Qin T. . (2022). The emerging roles of human gut microbiota in gastrointestinal Cancer. Front. Immunol. 13:5047. doi: 10.3389/fimmu.2022.915047, PMID: 35784372 PMC9240199

[ref37] Guo Y. Zhang Y. Gerhard M. Gao J. Mejias-Luque R. Zhang L. . (2020). Effect of *Helicobacter pylori* on gastrointestinal microbiota: a population-based study in Linqu, a high-risk area of gastric cancer. Gut 69, 1598–1607. doi: 10.1136/gutjnl-2019-319696, PMID: 31857433 PMC7456744

[ref38] He C. Peng C. Xu X. Li N. Ouyang Y. Zhu Y. . (2022). Probiotics mitigate *Helicobacter pylori*-induced gastric inflammation and premalignant lesions in INS-GAS mice with the modulation of gastrointestinal microbiota. Helicobacter 27:e12898. doi: 10.1111/hel.12898, PMID: 35531615

[ref39] He Z. Tian W. Wei Q. Xu J. (2022). Involvement of *Fusobacterium nucleatum* in malignancies except for colorectal cancer: a literature review. Front. Immunol. 13:649. doi: 10.3389/fimmu.2022.968649, PMID: 36059542 PMC9428792

[ref40] Hsieh Y. Kuo W. Hsu W. Tung S. Li C. (2023). *Fusobacterium Nucleatum*-induced tumor mutation burden predicts poor survival of gastric Cancer patients. Cancers 15:269. doi: 10.3390/cancers15010269, PMID: 36612265 PMC9818776

[ref41] Hsieh Y. Tung S. Pan H. Chang T. Wei K. Chen W. . (2021). *Fusobacterium nucleatum* colonization is associated with decreased survival of *helicobacter pylori*-positive gastric cancer patients. World J. Gastroenterol. 27, 7311–7323. doi: 10.3748/wjg.v27.i42.7311, PMID: 34876791 PMC8611209

[ref42] Hsieh Y. Tung S. Pan H. Yen C. Xu H. Lin Y. . (2018). Increased abundance of Clostridium and Fusobacterium in gastric microbiota of patients with gastric Cancer in Taiwan. Sci. Rep. 8:158. doi: 10.1038/s41598-017-18596-0, PMID: 29317709 PMC5760541

[ref43] Hu Y. Xu X. Ouyang Y. He C. Li N. Xie C. . (2022). Optimization of vonoprazan-amoxicillin dual therapy for eradicating *Helicobacter pylori*infection in China: a prospective, randomized clinical pilot study. Helicobacter 27:e12896. doi: 10.1111/hel.12896, PMID: 35466521

[ref44] Iino C. Shimoyama T. (2021). Impact of *Helicobacter pylori* infection on gut microbiota. World J. Gastroenterol. 27, 6224–6230. doi: 10.3748/wjg.v27.i37.6224, PMID: 34712028 PMC8515792

[ref45] Imai S. Ooki T. Murata-Kamiya N. Komura D. Tahmina K. Wu W. . (2021). *Helicobacter pylori* CagA elicits BRCAness to induce genome instability that may underlie bacterial gastric carcinogenesis. Cell Host Microbe 29, 941–958.e10. doi: 10.1016/j.chom.2021.04.006, PMID: 33989515

[ref46] Jiang K. Jiang X. Wen Y. Liao L. Liu F. (2019). Relationship between long-term use of proton pump inhibitors and risk of gastric cancer: a systematic analysis. J. Gastroenterol. Hepatol. 34, 1898–1905. doi: 10.1111/jgh.14759, PMID: 31206764

[ref47] Kakiuchi T. Matsuo M. Endo H. Sakata Y. Esaki M. Noda T. . (2023). Efficacy and safety of vonoprazan-based regimen for *Helicobacter pylori* eradication therapy in Japanese adolescents: a prospective multicenter study. J. Gastroenterol. 58, 196–204. doi: 10.1007/s00535-022-01942-z, PMID: 36528706

[ref48] Kokol P. Blazun Vosner H. Zavrsnik J. (2021). Application of bibliometrics in medicine: a historical bibliometrics analysis. Health Info. Libr. J. 38, 125–138. doi: 10.1111/hir.12295, PMID: 31995273

[ref49] Kokol P. Kokol M. Zagoranski S. (2022). Machine learning on small size samples: a synthetic knowledge synthesis. Sci. Prog. 105:003685042110297. doi: 10.1177/00368504211029777, PMID: 35220816 PMC10358596

[ref50] Kuipers E. J. (1998). Review article: relationship between *Helicobacter pylori*, atrophic gastritis and gastric cancer. Aliment. Pharmacol. Ther. 12, 25–36. doi: 10.1111/j.1365-2036.1998.00009.x 9701002

[ref51] Kuipers E. J. Lundell L. KlinkenbergKnol E. C. Havu N. Festen H. Liedman B. . (1996). Atrophic gastritis and *Helicobacter pylori* infection in patients with reflux esophagitis treated with omeprazole or fundoplication. N. Engl. J. Med. 334, 1018–1022. doi: 10.1056/NEJM199604183341603 8598839

[ref52] Kwon S. Park J. C. Kim K. H. Yoon J. Cho Y. Lee B. . (2022). Human gastric microbiota transplantation recapitulates premalignant lesions in germ-free mice. Gut 71, 1266–1276. doi: 10.1136/gutjnl-2021-324489, PMID: 34389621

[ref53] Lee S. A. Liu F. Riordan S. M. Lee C. S. Zhang L. (2019). Global investigations of *Fusobacterium nucleatum* in human colorectal Cancer. Front. Oncol. 9:566. doi: 10.3389/fonc.2019.00566, PMID: 31334107 PMC6618585

[ref54] Lehr K. Nikitina D. Vilchez-Vargas R. Steponaitiene R. Thon C. Skieceviciene J. . (2023). Microbial composition of tumorous and adjacent gastric tissue is associated with prognosis of gastric cancer. Sci. Rep. 13:4640. doi: 10.1038/s41598-023-31740-3, PMID: 36944721 PMC10030820

[ref55] Lei R. Zhang J. Zhou H. Liu Q. Wang H. Liu J. (2020). Analysis of population health projects funded by joint Fund of the National Natural Science Foundation of China between 2015 and 2019. Annals Transl Med 8:1477. doi: 10.21037/atm-20-6495, PMID: 33313222 PMC7729363

[ref56] Li Y. Choi H. Leung K. Jiang F. Graham D. Y. Leung W. K. (2023). Global prevalence of *Helicobacter pylori* infection between 1980 and 2022: a systematic review and meta-analysis. Lancet Gastroenterol. Hepatol. 8, 553–564. doi: 10.1016/S2468-1253(23)00070-5, PMID: 37086739

[ref57] Liao O. Ye G. Du Q. Ye J. (2023). Gastric microbiota in gastric cancer and precancerous stages: mechanisms of carcinogenesis and clinical value. Helicobacter 28:e12964. doi: 10.1111/hel.12964, PMID: 36880502

[ref58] Liu Q. Chen C. He Y. Mai W. Ruan S. Ning Y. . (2023). Notch signaling regulates the function and phenotype of dendritic cells in *Helicobacter pylori* infection. Microorganisms 11:2818. doi: 10.3390/microorganisms11112818, PMID: 38004829 PMC10673485

[ref59] Liu C. Ng S. K. Ding Y. Lin Y. Liu W. Wong S. H. . (2022). Meta-analysis of mucosal microbiota reveals universal microbial signatures and dysbiosis in gastric carcinogenesis. Oncogene 41, 3599–3610. doi: 10.1038/s41388-022-02377-9, PMID: 35680985 PMC9270228

[ref60] Lofgren J. L. Whary M. T. Ge Z. Muthupalani S. Taylor N. S. Mobley M. . (2011). Lack of commensal Flora in *Helicobacter pylori*-infected INS-GAS mice reduces gastritis and delays intraepithelial neoplasia. Gastroenterology 140, 210–220.e4. doi: 10.1053/j.gastro.2010.09.048, PMID: 20950613 PMC3006487

[ref61] Marshall B. (2023). Epidemiology of Helicobacter in Chinese families: a foundation for cost-effective eradication strategies? Gut:29:gutjnl-2023-329786. doi: 10.1136/gutjnl-2023-329786, PMID: 36990678 PMC11041556

[ref62] McCarthy D. M. (2020). Proton pump inhibitor use, Hypergastrinemia, and gastric carcinoids-what is the relationship? Int. J. Mol. Sci. 21:662. doi: 10.3390/ijms21020662, PMID: 31963924 PMC7014182

[ref63] Moral-Munoz J. A. Herrera-Viedma E. Santisteban-Espejo A. Cobo M. J. (2020). Software tools for conducting bibliometric analysis in science: an up-to-date review. Profesional e La Informacion 29:3. doi: 10.3145/epi.2020.ene.03

[ref64] Murata-Kamiya N. Hatakeyama M. (2022). *Helicobacter pylori*-induced DNA double-stranded break in the development of gastric cancer. Cancer Sci. 113, 1909–1918. doi: 10.1111/cas.15357, PMID: 35359025 PMC9207368

[ref65] Musazadeh V. Nazari A. Faghfouri A. H. Emami M. Kavyani Z. Zokaei M. . (2023). The effectiveness of treatment with probiotics in *Helicobacter pylori* eradication: −−/esults from an umbrella meta-analysis on meta-analyses of randomized controlled trials. Food Funct. 14, 7654–7662. doi: 10.1039/d3fo00300k, PMID: 37540067

[ref66] Nasr R. Shamseddine A. Mukherji D. Nassar F. Temraz S. (2020). The crosstalk between microbiome and immune response in gastric Cancer. Int. J. Mol. Sci. 21:6586. doi: 10.3390/ijms21186586 32916853 PMC7556019

[ref67] Nie S. Wang A. Yuan Y. (2021). Comparison of clinicopathological parameters, prognosis, micro-ecological environment and metabolic function of gastric Cancer with or without Fusobacterium sp. infection. J. Cancer 12, 1023–1032. doi: 10.7150/jca.50918, PMID: 33442401 PMC7797643

[ref68] Niu Z. Li S. Shi Y. Xue Y. (2021). Effect of gastric microbiota on quadruple *Helicobacter pylori* eradication therapy containing bismuth. World J. Gastroenterol. 27, 3913–3924. doi: 10.3748/wjg.v27.i25.3913, PMID: 34321854 PMC8291010

[ref69] Oster P. Vaillant L. McMillan B. Velin D. (2022a). The efficacy of Cancer immunotherapies is compromised by *Helicobacter pylori* infection. Front. Immunol. 13:161. doi: 10.3389/fimmu.2022.899161, PMID: 35677057 PMC9168074

[ref70] Oster P. Vaillant L. Riva E. McMillan B. Begka C. Truntzer C. . (2022b). *Helicobacter pylori* infection has a detrimental impact on the efficacy of cancer immunotherapies. Gut 71, 457–466. doi: 10.1136/gutjnl-2020-323392, PMID: 34253574 PMC8862014

[ref71] Ouyang Y. Wang M. Xu Y. Zhu Y. Lu N. Hu Y. (2022). Amoxicillin-vonoprazan dual therapy for *Helicobacter pylori* eradication: a systematic review and meta-analysis. J. Gastroenterol. Hepatol. 37, 1666–1672. doi: 10.1111/jgh.15917, PMID: 35716370

[ref72] Owyang S. Y. Zhang M. El-Zaatari M. Eaton K. A. Bishu S. Hou G. . (2020). Dendritic cell-derived TGF-β mediates the induction of mucosal regulatory T-cell response to Helicobacter infection essential for maintenance of immune tolerance in mice. Helicobacter 25:e12763. doi: 10.1111/hel.12763, PMID: 33025641 PMC7885176

[ref73] Png C. W. Lee W. J. J. Chua S. J. Zhu F. Yeoh K. G. Zhang Y. (2022). Mucosal microbiome associates with progression to gastric cancer. Theranostics 12, 48–58. doi: 10.7150/thno.65302, PMID: 34987633 PMC8690935

[ref74] Qi J. Li M. Wang L. Hu Y. Liu W. Long Z. . (2023). National and subnational trends in cancer burden in China, 2005-20: an analysis of national mortality surveillance data. Lancet Public Health 8, E943–E955. doi: 10.1016/S2468-2667(23)00211-6, PMID: 38000889

[ref75] Raspe J. Contoyannis A. Schmitz M. Uebner H. Cover T. Mueller A. . (2023). *Helicobacter pylori* protein vacuolating Cytotoxin a (VacA) modulates the murine and human interaction between dendritic cells and T cells. Pneumologie 771:S71. doi: 10.1055/s-0043-1761034

[ref76] Ravikumara M. (2023). *Helicobacter pylori* in children: think before you kill the bug! Therap. Adv. Gastroenterol. 16:610. doi: 10.1177/17562848231177610, PMID: 37361453 PMC10285598

[ref77] Rubinstein M. R. Baik J. E. Lagana S. M. Han R. P. Raab W. J. Sahoo D. . (2019). *Fusobacterium nucleatum* promotes colorectal cancer by inducing Wnt/β-catenin modulator Annexin A1. EMBO Rep. 20:638. doi: 10.15252/embr.201847638, PMID: 30833345 PMC6446206

[ref78] Rubinstein M. R. Wang X. Liu W. Hao Y. Cai G. Han Y. W. (2013). *Fusobacterium nucleatum* promotes colorectal carcinogenesis by modulating E-cadherin/β-catenin signaling via its FadA Adhesin. Cell Host Microbe 14, 195–206. doi: 10.1016/j.chom.2013.07.012, PMID: 23954158 PMC3770529

[ref79] Rugge M. Meggio A. Pravadelli C. Barbareschi M. Fassan M. Gentilini M. . (2019). Gastritis staging in the endoscopic follow-up for the secondary prevention of gastric cancer: a 5-year prospective study of 1755 patients. Gut 68, 11–17. doi: 10.1136/gutjnl-2017-314600, PMID: 29306868

[ref80] Seo S. I. Park C. H. You S. C. Kim J. Y. Lee K. J. Kim J. . (2021). Association between proton pump inhibitor use and gastric cancer: a population-based cohort study using two different types of nationwide databases in Korea. Gut 70, 2066–2075. doi: 10.1136/gutjnl-2020-323845, PMID: 33975868

[ref81] Seol M. Lee Y. R. Kim K. M. Shin C. M. Yoon H. Cho J. H. . (2019). The difference of the gut microbiota of gastric cancer in relation to *Helicobacter pylori* negativity and positivity. J. Clin. Oncol. 37S. doi: 10.1200/JCO.2019.37.4_suppl.10

[ref82] Singh V. Singh H. Dhiman B. Kumar N. Singh T. (2023). Analyzing bibliometric and thematic patterns in the transition to sustainable transportation: uncovering the influences on electric vehicle adoption. Res. Transp. Bus. Manag. 50:101033. doi: 10.1016/j.rtbm.2023.101033

[ref83] Sonnenberg A. (2022). Epidemiology of *Helicobacter pylori*. Aliment. Pharmacol. Ther. 55, S1–S13. doi: 10.1111/apt.16592 34989430

[ref84] Sung J. Coker O. O. Chu E. Szeto C. H. Luk S. Lau H. . (2020). Gastric microbes associated with gastric inflammation, atrophy and intestinal metaplasia 1 year after *Helicobacter pylori* eradication. Gut 69, 1572–1581. doi: 10.1136/gutjnl-2019-319826, PMID: 31974133 PMC7456733

[ref85] Sung H. Ferlay J. Siegel R. L. Laversanne M. Soerjomataram I. Jemal A. . (2021). Global Cancer statistics 2020: GLOBOCAN estimates of incidence and mortality worldwide for 36 cancers in 185 countries. CA Cancer J. Clin. 71, 209–249. doi: 10.3322/caac.21660, PMID: 33538338

[ref86] Suzuki S. Kusano C. Horii T. Ichijima R. Ikehara H. (2022). The ideal *Helicobacter pylori* treatment for the present and the future. Digestion 103, 62–68. doi: 10.1159/000519413, PMID: 34662879

[ref87] Takahashi-Kanemitsu A. Knight C. T. Hatakeyama M. (2020). Molecular anatomy and pathogenic actions of *Helicobacter pylori* CagA that underpin gastric carcinogenesis. Cell. Mol. Immunol. 17, 50–63. doi: 10.1038/s41423-019-0339-5, PMID: 31804619 PMC6952403

[ref88] Teng Y. Xia C. Li H. Cao M. Yang F. Yan X. . (2023). Cancer statistics for young adults aged 20 to 49 years in China from 2000 to 2017: a population-based registry study. Sci. China Life Sci. doi: 10.1007/s11427-023-2445-1 38155276

[ref89] van Eck N. J. Waltman L. (2010). Software survey: VOSviewer, a computer program for bibliometric mapping. Scientometrics 84, 523–538. doi: 10.1007/s11192-009-0146-3, PMID: 20585380 PMC2883932

[ref90] Viazis N. Argyriou K. Kotzampassi K. Christodoulou D. K. Apostolopoulos P. Georgopoulos S. D. . (2022). A four-probiotics regimen combined with a standard *Helicobacter pylori*-eradication treatment reduces side effects and increases eradication rates. Nutrients 14:632. doi: 10.3390/nu14030632, PMID: 35276991 PMC8838490

[ref91] Wang Z. Han W. Xue F. Zhao Y. Wu P. Chen Y. . (2022). Nationwide gastric cancer prevention in China, 2021-2035: a decision analysis on effect, affordability and cost-effectiveness optimisation. Gut 71, 2391–2400. doi: 10.1136/gutjnl-2021-325948, PMID: 35902213

[ref92] Wang X. Wang L. Xu L. Liang S. Yu M. Zhang Q. . (2023). Evaluation of polygenic risk score for risk prediction of gastric cancer. World J Gastrointest Oncol 15, 276–285. doi: 10.4251/wjgo.v15.i2.276, PMID: 36908320 PMC9994049

[ref93] Wong B. C. Lam S. K. Wong W. M. Chen J. S. Zheng T. T. Feng R. E. . (2004). *Helicobacter pylori* eradication to prevent gastric cancer in a high-risk region of China: a randomized controlled trial. JAMA 291, 187–194. doi: 10.1001/jama.291.2.187, PMID: 14722144

[ref94] Xin Y. Li X. Zhang M. Shang Z. Luo Z. Wang Y. . (2023). *Fusobacterium nucleatum*-induced exosomal HOTTIP promotes gastric cancer progression through the microRNA-885-3p/EphB2 axis. Cancer Sci. 114, 2360–2374. doi: 10.1111/cas.15781, PMID: 36898846 PMC10236632

[ref95] Xu W. Chen G. Shao Y. Li X. Xu H. Zhang H. . (2013). Gastrin acting on the cholecystokinin2 receptor induces cyclooxygenase-2 expression through JAK2/STAT3/PI3K/Akt pathway in human gastric cancer cells. Cancer Lett. 332, 11–18. doi: 10.1016/j.canlet.2012.12.030, PMID: 23376640

[ref96] Xu C. Fan L. Lin Y. Shen W. Qi Y. Zhang Y. . (2021). *Fusobacterium nucleatum* promotes colorectal cancer metastasis through miR-1322/CCL20 axis and M2 polarization. Gut Microbes 13:347. doi: 10.1080/19490976.2021.1980347, PMID: 34632963 PMC8510564

[ref97] Yang J. Liu X. Cheng Y. Zhang J. Ji F. Ling Z. (2022). Roles of Plasmacytoid dendritic cells in gastric Cancer. Front. Oncol. 12:8314. doi: 10.3389/fonc.2022.818314, PMID: 35311157 PMC8927765

[ref98] Yang J. Lu C. Lin C. (2014). Treatment of *Helicobacter pylori* infection: current status and future concepts. World J. Gastroenterol. 20, 5283–5293. doi: 10.3748/wjg.v20.i18.5283, PMID: 24833858 PMC4017043

[ref99] Yuan X. Zhang Y. Zhao X. Chen A. Liu P. (2023). IL-1β, an important cytokine affecting *Helicobacter pylori*-mediated gastric carcinogenesis. Microb. Pathog. 174:105933. doi: 10.1016/j.micpath.2022.105933, PMID: 36494022

[ref100] Yue K. Sheng D. Xue X. Zhao L. Zhao G. Jin C. . (2023). Bidirectional mediation effects between Intratumoral microbiome and host DNA methylation changes contribute to stomach adenocarcinoma. Microbiol Spectr 11:e0090423. doi: 10.1128/spectrum.00904-23, PMID: 37260411 PMC10434028

[ref101] Zhang Z. Chen S. Fan M. Ruan G. Xi T. Zheng L. . (2021). *Helicobacter pylori* induces gastric cancer via down-regulating miR-375 to inhibit dendritic cell maturation. Helicobacter 26:e12813. doi: 10.1111/hel.12813, PMID: 33938607

[ref102] Zhang J. Hu C. Zhang R. Xu J. Zhang Y. Yuan L. . (2023). The role of macrophages in gastric cancer. Front. Immunol. 14:2176. doi: 10.3389/fimmu.2023.1282176, PMID: 38143746 PMC10746385

[ref103] Zhang W. Lin B. Li Y. Ding Y. Han Z. Ji R. (2023). Efficacy and safety of Vonoprazan and amoxicillin dual therapy for *Helicobacter pylori* eradication: a systematic review and Meta-analysis. Digestion 104, 249–261. doi: 10.1159/000529622, PMID: 37015201 PMC10407836

[ref104] Zhang J. Yu Q. Zheng F. Long C. Lu Z. Duan Z. (2016). Comparing keywords plus of WOS and author keywords: a case study of patient adherence research. J. Assoc. Inf. Sci. Technol. 67, 967–972. doi: 10.1002/asi.23437

[ref105] Zhang L. Zhao M. Fu X. (2023). Gastric microbiota dysbiosis and *Helicobacter pylori* infection. Front. Microbiol. 14:3269. doi: 10.3389/fmicb.2023.1153269, PMID: 37065152 PMC10098173

[ref106] Zhou T. Meng X. Wang D. Fu W. Li X. (2022). Neutrophil transcriptional deregulation by the periodontal pathogen *Fusobacterium nucleatum* in gastric Cancer: a Bioinformatic study. Dis. Markers 2022, 9584507–9584510. doi: 10.1155/2022/9584507, PMID: 36033825 PMC9410804

[ref107] Zhou H. Yang X. Liu Q. Pu J. Lei R. (2021). Distribution of the population and health projects of the joint Fund in China between 2006 and 2019. Annals Transl Med 9:1388. doi: 10.21037/atm-21-4364, PMID: 34733940 PMC8506539

[ref108] Zhu Y. Zhu F. Ba H. Chen J. Bian X. (2023). *Helicobacter pylori* infection and PD-L1 expression in gastric cancer: a meta-analysis. Eur. J. Clin. Invest. 53:3880. doi: 10.1111/eci.13880 36164962

